# Supplementary material for: Predicting and improving complex beer flavor through machine learning
Source: Nat Commun. 2024 Mar 26;15:2368. doi: 10.1038/s41467-024-46346-0 (PMC10966102; doi:10.1038/s41467-024-46346-0)
Supplement: Supplementary file 1 — Supplementary Information [file 41467_2024_46346_MOESM1_ESM.pdf]

SUPPLEMENTARY INFORMATION

Predicting and improving complex beer flavor through machine learning

Michiel Schreurs[1,2,3,*], Supinya Piampongsant[1,2,3,*], Miguel Roncoroni[1,2,3,*] , Lloyd Cool[1,2,3,4], Beatriz Herrera-Malaver[1,2,3], Christophe Vanderaa[4], Florian A. Theßeling[1,2,3], Łukasz Kreft[5], Alexander Botzki[5], Philippe Malcorps[6], Luk Daenen[6], Tom Wenseleers[4] and Kevin J. Verstrepen[1,2,3,°]

1 VIB – KU Leuven Center for Microbiology, Gaston Geenslaan 1, B-3001 Leuven, Belgium
2 CMPG Laboratory of Genetics and Genomics, KU Leuven, Gaston Geenslaan 1, B-3001 Leuven, Belgium
3 Leuven Institute for Beer Research (LIBR), Gaston Geenslaan 1, B-3001 Leuven, Belgium
4 Laboratory of Socioecology and Social Evolution, KU Leuven, Naamsestraat 59, B-3000 Leuven, Belgium
5 VIB Bioinformatics Core, VIB, Rijvisschestraat 120, B-9052 Ghent, Belgium
6 AB InBev SA/NV, Brouwerijplein 1, Leuven, Belgium
* Equal Contribution
° Corresponding author, e-mail: kevin.verstrepen@kuleuven.be

SUPPLEMENTARY FIGURES AND TABLES

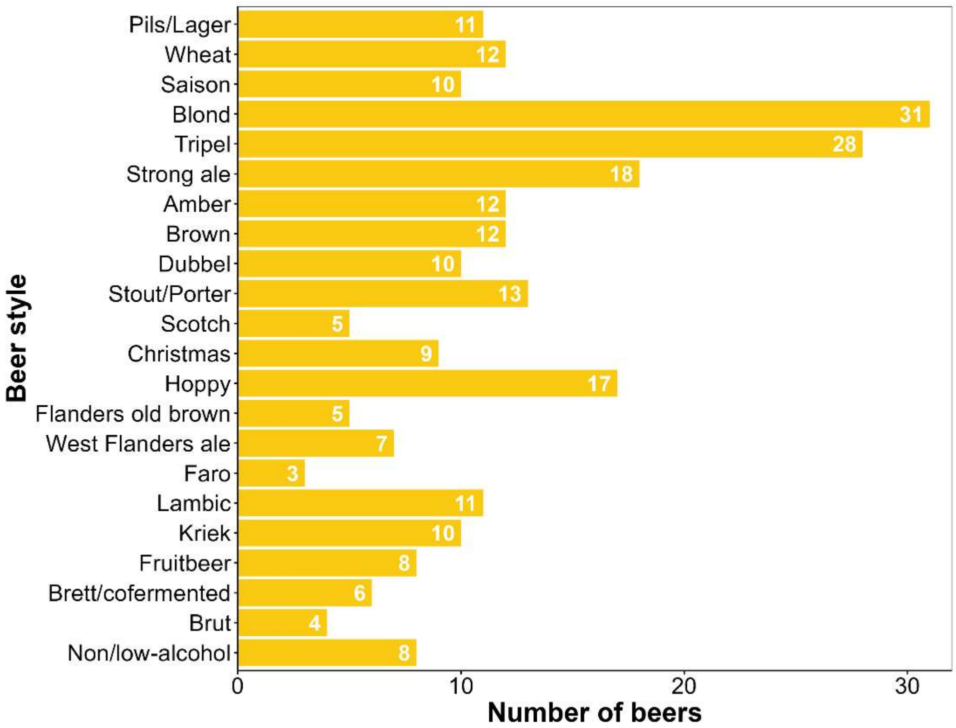

Supplementary Figure S1: The different beer styles under study. The corresponding number of beers is indicated per beer style (n = 250).

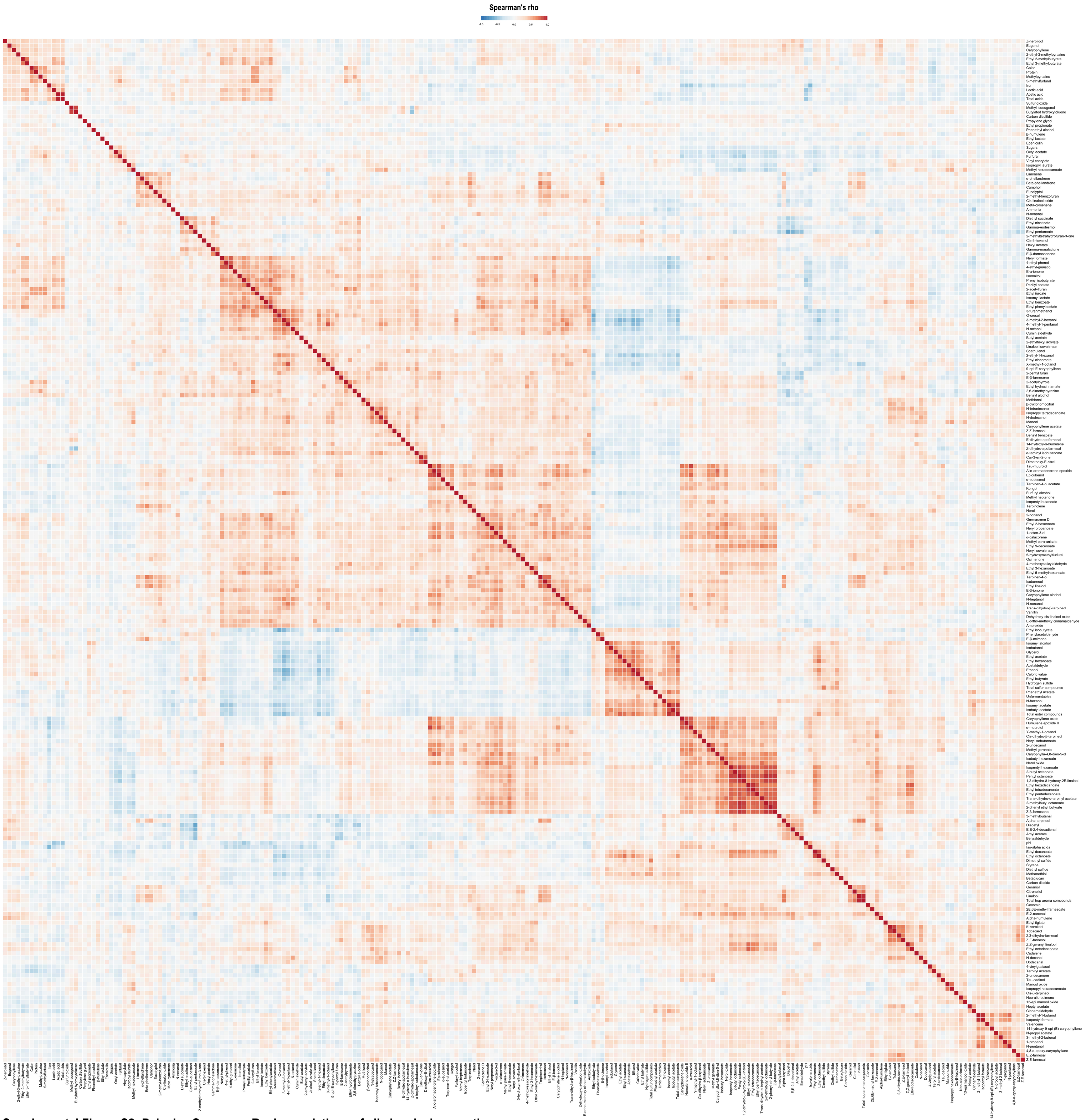

Supplemental Figure S2: Pairwise Spearman Rank correlations of all chemical properties.

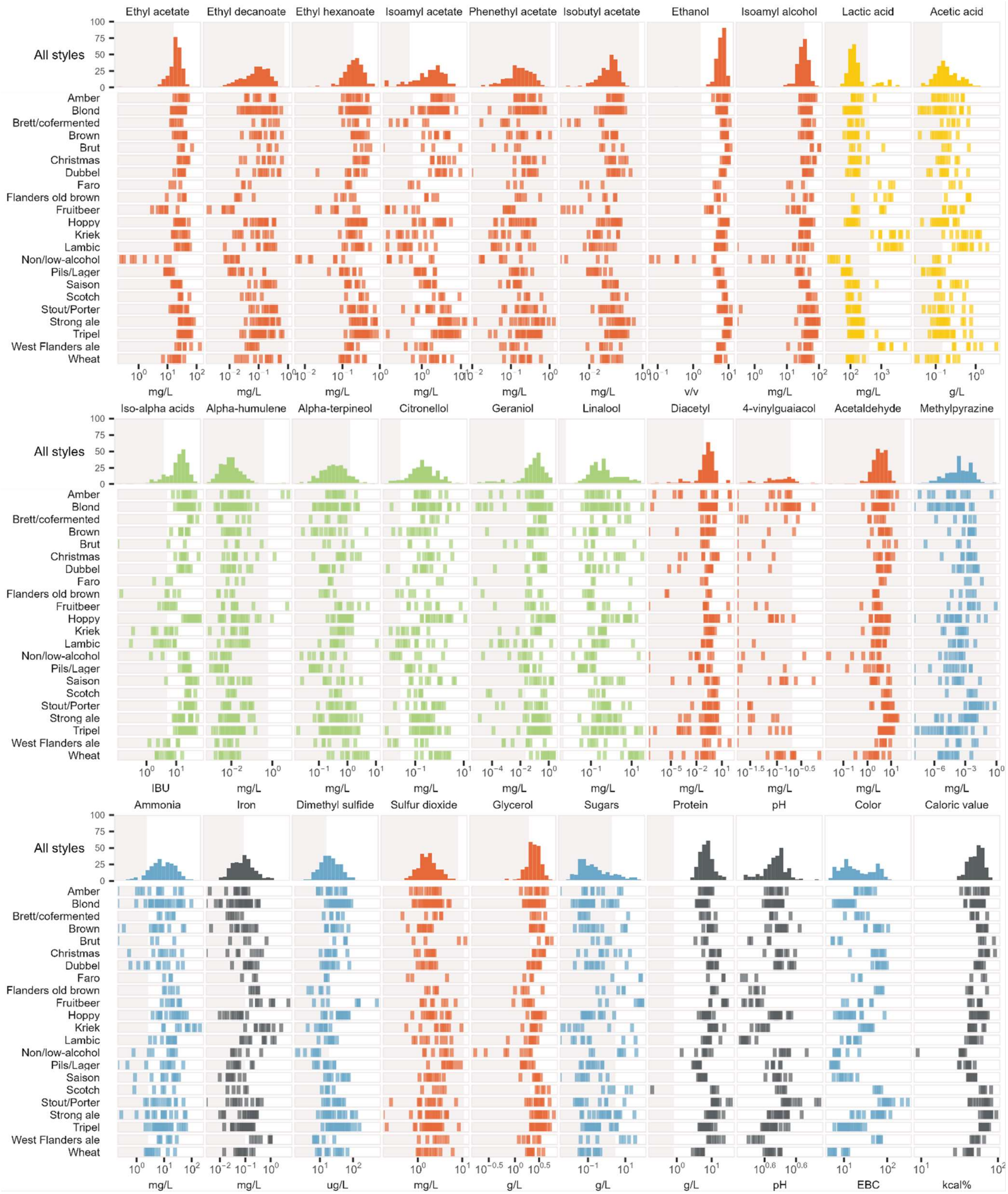

Supplementary Figure S3: Distributions of selected chemical properties across beer styles (n = 250 beers). Properties are colored according to their primary origin (*S. cerevisiae* (red), malt (blue), hops (green), other yeasts and bacteria (yellow), miscellaneous (black)). Compounds that are log-normal distributed (Shapiro-Wilk test $p < 0.05$) are represented on a log scale. Grey-shaded areas represent values below median reported taste thresholds.

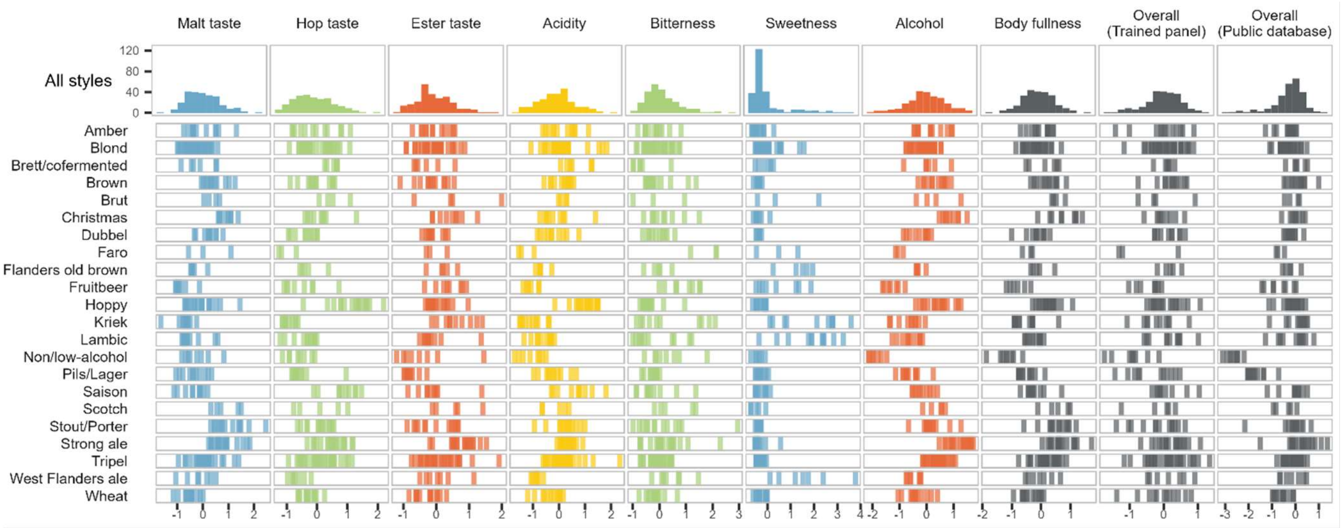

Supplementary Figure S4: Distributions of major sensory attributes per style (n = 250 beers). Each line represents the value of a single beer. Properties are colored according to their primary origin (*S. cerevisiae* (red), malt (blue), hops (green), other yeasts and bacteria (yellow), miscellaneous (black)).

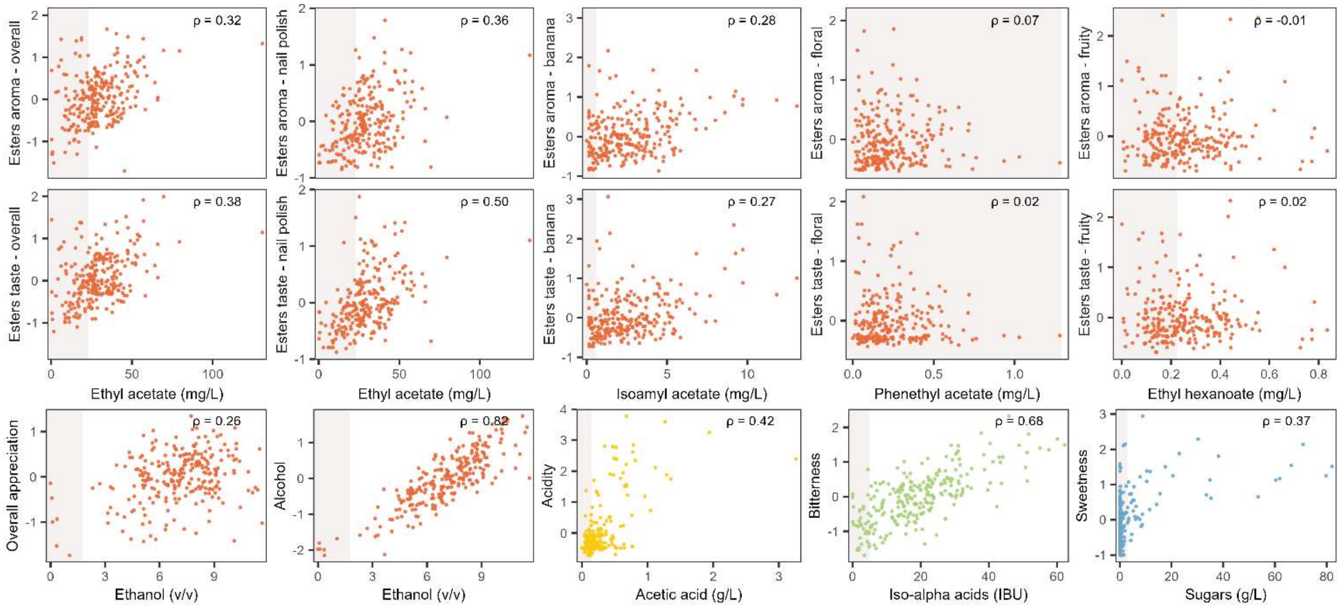

Supplementary Figure S5: Scatter plots of flavors vs. causative compounds (n = 250 beers). Grey-shaded areas represent values below median reported taste thresholds[1]. Rho values are Spearman's rank correlations. Dots are colored according to their primary origin (*S. cerevisiae* (red), malt (blue), hops (green), other yeasts and bacteria (yellow)).

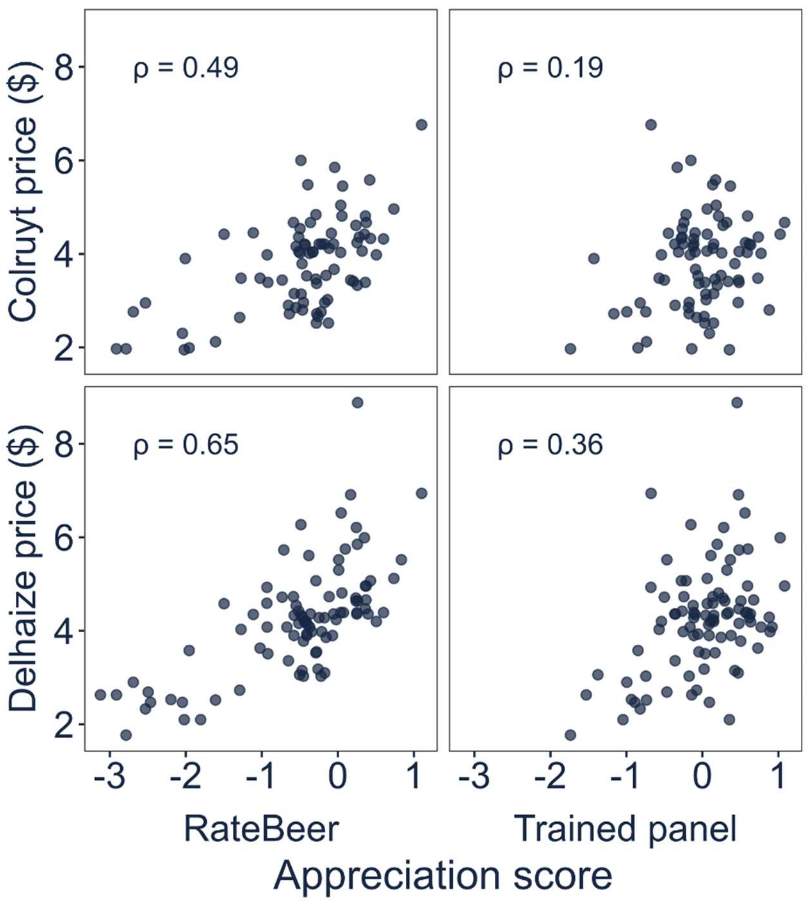

Supplementary Figure S6. Correlation between beer price and appreciation scores from our Trained Panel and RateBeer. Rho values shown are Spearman correlation coefficients. Beer prices were taken from the two Belgian food retail market leaders (Delhaize and Colruyt)[2]. The Delhaize dataset contains price information for 91 beers, the Colruyt dataset for 80 beers. Note that most online and US-based shops had extreme price outliers (mainly for specialty beers) which is why they were excluded from this analysis. It should be noted that expensive beers are overrepresented in the Delhaize dataset, and these expensive specialty beers are driving the minor price-appreciation correlation observed for our trained tasting panel.

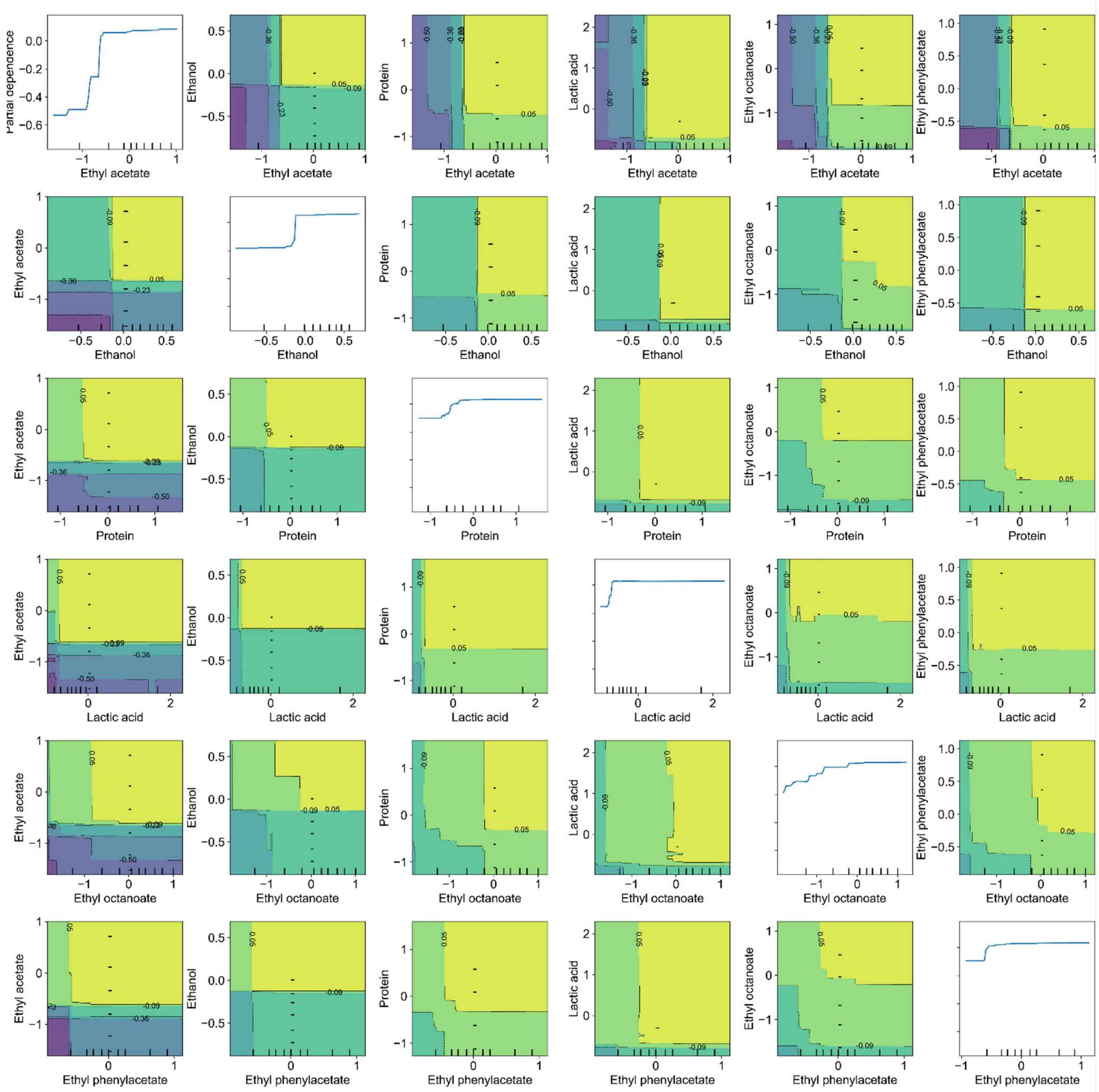

Supplementary Figure S7: Partial dependence plots of the most important parameters in the GBR model that predicts public appreciation scores from chemical profiles. One-way partial dependence plots are shown on the diagonal, with the corresponding two-way plots making up the rest of the figure. One-way partial dependence plots show how changing concentration of the chemical parameter (x-axis) influences the predicted appreciation (y-axis). Two-way plots combine two chemical compounds (x and y-axis), showing how a simultaneous change in concentration influences the predicted appreciation (color gradient, dark/purple: lower appreciation, bright/yellow: higher appreciation).

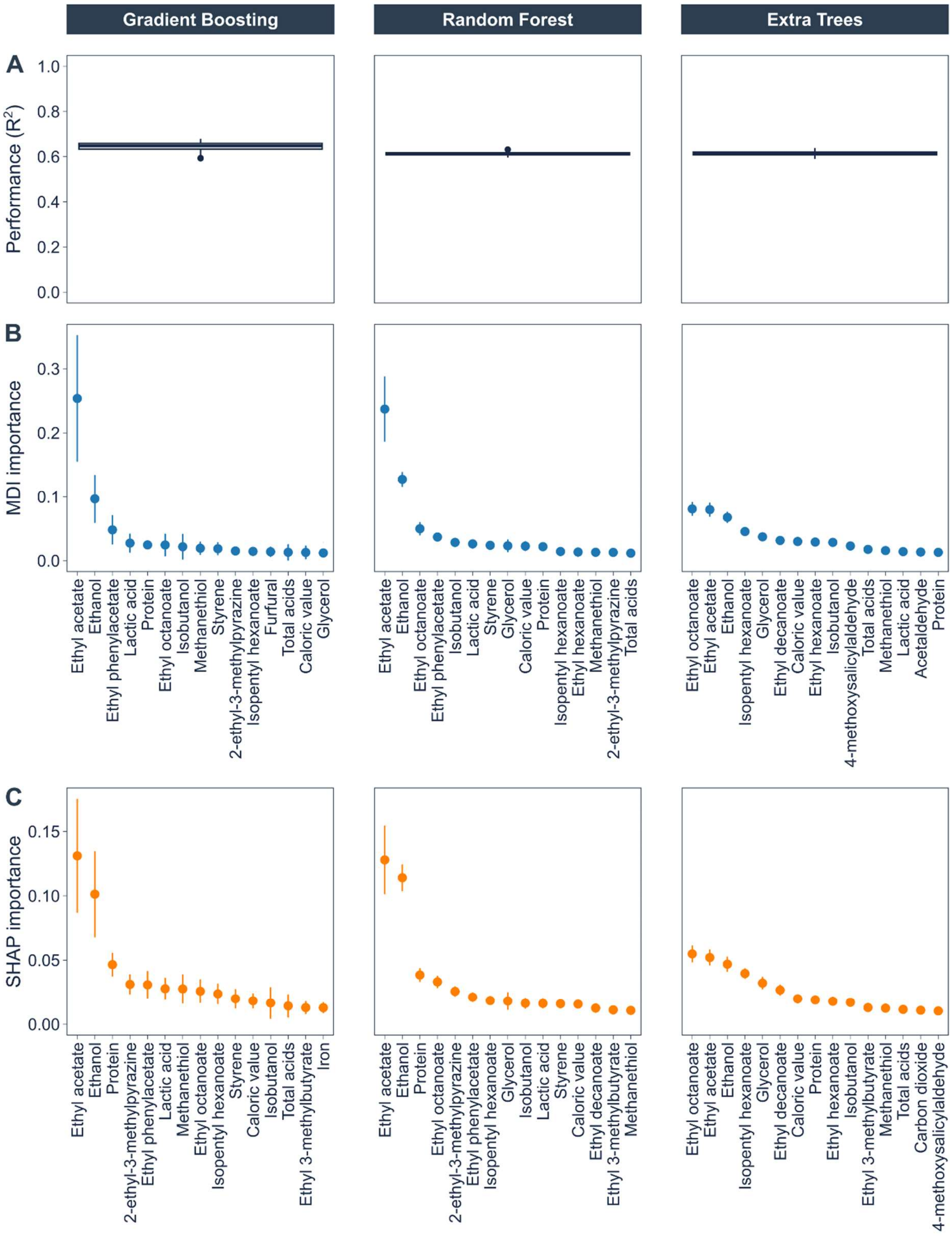

Supplementary Figure S8: Results from 100 random iterations of the Gradient Boosting, Random Forest and Extra Trees Regressor. A Boxplot showing the performance of the different models, measured by the R^2 metric (center line, median; box limits, upper and lower quartiles; whiskers, 1.5x interquartile range; points, outliers). **B** Average feature importance, calculated as Mean Decrease in Impurity (MDI). Error bars indicate the standard deviation around the mean. **C** Average feature importance, calculated with SHAP. Error bars indicate the standard deviation around the mean.

A

B

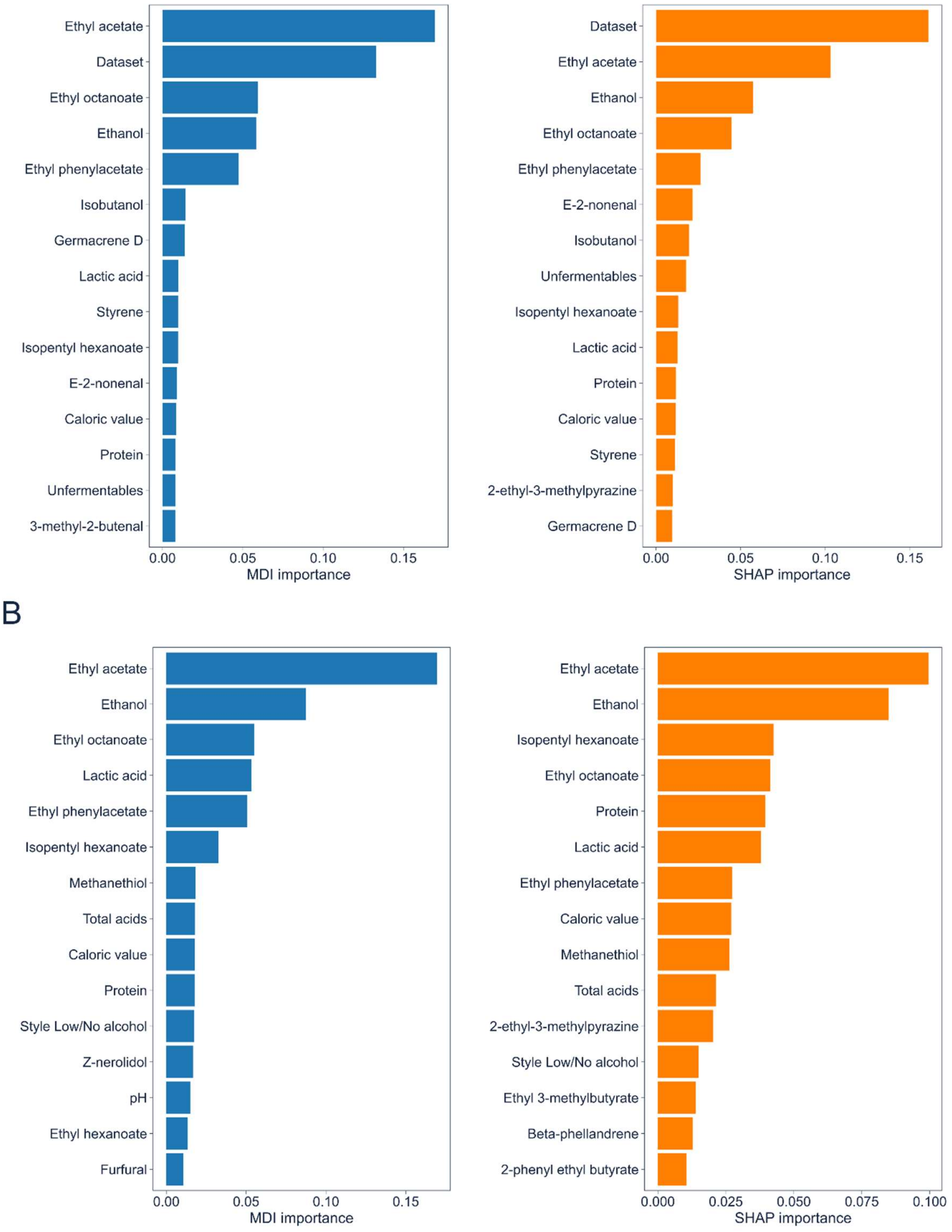

Supplementary Figure S9: A Feature importance (MDI and SHAP) of a model trained on both the RateBeer and Trained panel appreciation data. An identifier that encodes the datasets is considered highly important, indicating that the model tries to isolate the datasets before making predictions. Features that are more important for predicting trained panel appreciation, like E-2-nonenal and unfermentables, now appear in the top 15. **B Feature importance (MDI and SHAP) of a model trained with a beer style identifier.** Besides the inclusion of the ”no/low alcohol” style, which is appreciated the least of all the styles on average, the top 15 most important features remain largely the same.

Supplementary Table S1: List of measured chemical properties, their origin during the brewing process, the method used to determine their levels and their perceived organoleptic characteristics, together with the reference. If no flavor information was found in literature, the cell is left blank.

Compound	Compound class	Origin	Method of detection	Flavor	Flavor reference
2-acetylfuran	Furan	Malt, Aging	GC-MS	almond, rubber, burnt	ASBC Beer flavor database
2-acetylpyrrole	Pyrrole	Malt	GC-MS	walnut, liquorice	ASBC Beer flavor database
acetaldehyde	Aldehyde	Yeast, Aging	GC-FID	Green apple, paint	ASBC Beer flavor database
acetic acid	Acid	Bacteria	Enzymatic assay	vinegar	ASBC Beer flavor database
allo-aromadendrene epoxide	Terpenoid	Hops	GC-MS		
ambroxide	Terpenoid	Spices	GC-MS		
ammonia	Inorganic	Malt	Enzymatic assay	sour	ASBC Beer flavor database
amyl acetate	Ester	Yeast	GC-MS	fruity, pear, banana	The Good Scents Company
2,3-butanedione	Ketone	Yeast, Aging	GC-MS	butterscotch, buttery	ASBC Beer flavor database The Good Scents Company
2-butyl octanoate	Ester	Yeast	GC-MS	buttery	The Good Scents Company
benzaldehyde	Aldehyde	Yeast, Aging	GC-MS	almond, fruity cherry	ASBC Beer flavor database The Good Scents Company
benzyl alcohol	Alcohol	Hops	GC-MS	almond, bitter, chemical fruity cherry balsamic	ASBC Beer flavor database The Good Scents Company
benzyl benzoate	Ester	Additive	GC-MS	balsamic	The Good Scents Company
betaglucan	Carbohydrate	Malt, Yeast	Enzymatic assay		
butyl acetate	Ester	Yeast	GC-MS	solvent-like, banana, acetone, sweet	The Good Scents Company
butylated hydroxytoluene	Phenol	Additive	GC-MS		
9-epi-E-caryophyllene	Terpenoid	Hops	GC-MS		
cadalene	Terpenoid	Hops	GC-MS		
Caloric value	Miscellaneous	Malt, Yeast	GC-MS		
camphor	Terpenoid	Spices	GC-MS	herbal, spicy, woody	PerfumersWorld
car-3-en-2-one	Terpenoid	Hops	GC-MS		
carbon dioxide	Acid	Yeast	Pressure measurement	sour	ASBC Beer flavor database
carbon disulfide	Sulfur compound	Yeast	GC-FPD		
caryophylla-4,8-dien-5-ol	Terpenoid	Hops	GC-MS		
caryophyllene acetate	Terpenoid	Hops	GC-MS	woody, spicy, cedar	The Good Scents Company
caryophyllene alcohol	Terpenoid	Hops	GC-MS	Spicy, moss, earthy	The Good Scents Company
caryophyllene oxide	Terpenoid	Hops	GC-MS	woody, cedar, spicy	The Good Scents Company
caryophyllene	Terpenoid	Hops	GC-MS	woody, spicy, floral	ASBC Beer flavor database
cinnamaldehyde	Aldehyde	Spices	GC-MS	cinnamon, sweet	ASBC Beer flavor database
citronellol	Terpenoid	Hops, Spices	GC-MS	floral, citrus, roses	ASBC Beer flavor database

color (EBC)	Miscellaneous	Malt	Spectro-photometric		
cuminaldehyde	Aldehyde	Spices	GC-MS	cumin seed	ASBC Beer flavor database
meta-cymenene	Terpenoid	Hops	GC-MS		
o-cresol	Phenol	Yeast	GC-MS	phenolic, medicinal	The Good Scents Company
tau-cadinol	Terpenoid	Hops	GC-MS		
α-calacorene	Terpenoid	Hops	GC-MS	woody	ASBC Hop flavor database
β-cyclohomocitral	Terpenoid	Hops	GC-MS		
1,2-dihydro-8-hydroxy-2E-linalool	Terpenoid	Hops	GC-MS		
2,3-dihydro-farnesol	Alcohol	Hops	GC-MS	floral, lily	The Good Scents Company
2,6-dimethylpyrazine	Pyrazine	Malt	GC-MS	ethereal, cocoa, nutty	The Good Scents Company
cis-dihydro-β-terpineol	Terpenoid	Hops	GC-MS		
dehydroxy-cis-linalool oxide	Terpenoid	Hops	GC-MS		
diethyl succinate	Ester	Aging, Yeast	GC-MS	Fruity, apples	The Good Scents Company
diethyl sulfide	Sulfur compound	Yeast	GC-FPD	cooked vegetables	ASBC Beer flavor database
dihydroisocaryophyllene epoxide	Terpenoid	Hops	GC-MS	woody, tabacco	Patent US3620982A
dimethoxy-E-citral	Terpenoid	Hops	GC-MS		
dimethyl sulfide	Sulfur compound	Bacteria, Aging	GC-FPD	cooked vegetables, corn, garlic	ASBC Beer flavor database
dodecanal	Aldehyde	Spices	GC-MS	caprylic, soapy, waxy	ASBC Beer flavor database The Good Scents Company
E,E-2,4-decadienal	Aldehyde	Aging, Yeast	GC-MS	oily, rancid, papery	ASBC Beer flavor database
E-dihydro-apofarnesal	Terpenoid	Hops	GC-MS		
E-β-damascenone	Ketone	Hops, Aging	GC-MS	apple, honey, fruity	ASBC Hop flavor database
n-decanol	Alcohol	Yeast	GC-MS	coconut, walnut, oily	ASBC Beer flavor database
n-dodecanol	Alcohol	Yeast	GC-MS	fatty, coconut, banana	ASBC Beer flavor database
trans-dihydro-α-terpinyl acetate	Terpenoid	Hops	GC-MS		
trans-dihydro-β-terpineol	Terpenoid	Spices, Hops	GC-MS		
Z-dihydro-apofarnesal	Terpenoid	Hops	GC-MS		
2-ethyl-1-hexanol	Alcohol	Yeast	GC-MS	citrus	The Good Scents Company
2-ethyl-3-methylpyrazine	Pyrazine	Malt	GC-MS	nutty , peanut	The Good Scents Company
2-ethylhexylsalicylate	Ester	Hops	GC-MS	floral	The Good Scents Company
4,8-α-epoxy-caryophyllene	Terpenoid	Hops	GC-MS		
4-ethyl-guaiacol	Phenol	Yeast, Wood	GC-MS	phenolic, medicinal, spicy, woody, bacon	ASBC Beer flavor database The Good Scents Company
4-ethyl-phenol	Phenol	Yeast, Wood	GC-MS	phenolic, smoky	ASBC Beer flavor database The Good Scents Company
epicubenol	Terpenoid	Hops	GC-MS		
ethanol	Alcohol	Yeast	Spectro-photometric	warming, strong, alcoholic	ASBC Beer flavor database
ethyl 2-hexenoate	Ester	Yeast	GC-MS		

ethyl 2-methylbutyrate	Ester	Yeast, Aging	GC-FID	fruity, apple, sweet	ASBC Beer flavor database
ethyl 3-hexanoate	Ester	Yeast	GC-MS		
ethyl 5-methylhexanoate	Ester	Yeast	GC-MS		
ethyl 9-decenoate	Ester	Yeast	GC-MS		
ethyl acetate	Ester	Yeast	GC-FID	solvent, fruity, sweet	ASBC Beer flavor database
ethyl benzoate	Ester	Yeast	GC-MS	fruity, sweet, wintergreen	The Good Scents Company
ethyl butyrate	Ester	Yeast	GC-MS	papaya, butter, sweet, fruity	ASBC Beer flavor database
ethyl cinnamate	Ester	Aging, Spices	GC-MS	fruity, sweet	ASBC Beer flavor database
ethyl decanoate	Ester	Yeast	GC-FID	caprylic, fruity, apple	ASBC Beer flavor database
ethyl furoate	Ester	Malt	GC-MS		
ethyl hexadecanoate	Ester	Yeast	GC-MS	fatty, fruity, sweet, rancid	ASBC Beer flavor database
ethyl hexanoate	Ester	Yeast	GC-FID	sour apple, aniseed, fruity, sweet	ASBC Beer flavor database
ethyl hydrocinnamate	Ester	Spices	GC-MS	floral	The Good Scents Company
ethyl isobutyrate	Ester	Yeast	GC-MS	apple, sweet, citrus, fruity	ASBC Beer flavor database
ethyl isovalerate	Ester	Yeast, Aging	GC-FID	fruity, apple, sweet	ASBC Beer flavor database
ethyl lactate	Ester	Yeast, Aging	GC-MS	strawberry, raspberry	ASBC Beer flavor database
ethyl linalool	Terpenoid	Hops	GC-MS	Bergamot, floral	The Good Scents Company
ethyl nicotinate	Ester	Yeast, Aging	GC-MS	grainy, medicinal, stale	ASBC Beer flavor database
ethyl octadecanoate	Ester	Yeast	GC-MS	fatty	ASBC Beer flavor database
ethyl octanoate	Ester	Yeast	GC-FID	Apple, fruity	ASBC Beer flavor database
ethyl pentadecanoate	Ester	Yeast	GC-MS	fatty	ASBC Beer flavor database
ethyl pentanoate	Ester	Yeast	GC-MS	papaya, fruity, apple, sweet	ASBC Beer flavor database
ethyl phenylacetate	Ester	Aging, Yeast	GC-MS	honey, roses, floral	The Good Scents Company
ethyl propionate	Ester	Yeast	GC-FID	fruity, sweet	The Good Scents Company
ethyl tetradecanoate	Ester	Yeast	GC-MS	fatty, caprylic	ASBC Beer flavor database
ethyl tiglate	Ester	Yeast	GC-MS	fruity, berry, tutti frutti	The Good Scents Company
eucalyptol	Terpenoid	Hops	GC-MS	herbal	The Good Scents Company
eugenol	Terpenoid	Spices, Yeast, Wood	GC-MS	clove, sweet, dentist	ASBC Beer flavor database
gamma-eudesmol	Terpenoid	Spicy	GC-MS	waxy, sweet	ASBC Beer flavor database
α-eudesmol	Terpenoid	Spices	GC-MS		
3-furanmethanol	Alcohol	Malt	GC-MS		
4-fluorobenzaldehyde	Aldehyde	Internal standard solution	GC-MS		
E,Z-farnesal	Terpenoid	Hops	GC-MS	floral, minty	The Good Scents Company

E-β-farnesene	Terpenoid	Hops	GC-MS	woody, citrus, sweet	ASBC Hop flavor database
foeniculin	Terpenoid	Spices	GC-MS		
furfural	Aldehyde	Malt, Aging, Wood	GC-MS	caramel, bread	ASBC Beer flavor database
Z,E-farnesal	Terpenoid	Hops	GC-MS	floral, minty	The Good Scents Company
Z,E-farnesol	Alcohol	Hops	GC-MS	lily	ASBC Hop flavor database
Z,Z-farnesol	Alcohol	Hops	GC-MS	lily	ASBC Hop flavor database
Z-β-farnesene	Terpenoid	Hops	GC-MS	woody, citrus, sweet	ASBC Hop flavor database
furfuryl alcohol	Alcohol	Aging	GC-MS	sugar cane, woody	ASBC Beer flavor database
geosmin	Terpenoid	Bacteria	GC-MS	earthy, humus	The Good Scents Company
geraniol	Terpenoid	Hops	GC-MS	floral, citrus, roses	ASBC Hop flavor database
germacrene D	Terpenoid	Hops	GC-MS	woody, spicy	The Good Scents Company
glycerol	Carbohydrate	Yeast	Enzymatic assay	sweet	The Good Scents Company
guaiacol	Phenol	Internal standard solution	GC-MS	smoky	ASBC Beer flavor database
Z,Z-geranyl linalool	Terpenoid	Hops	GC-MS		
14-hydroxy-9-epi-(E)-caryophyllene	Terpenoid	Spices	GC-MS		
14-hydroxy-α-humulene	Terpenoid	Hops	GC-MS		
2,3-hexanedione	Ketone	Internal standard solution	GC-MS	strawberry, butter	ASBC Beer flavor database
2-heptanol	Alcohol	Internal standard solution	GC-FID/GC-MS	coconut	ASBC Beer flavor database
5-hydroxymethylfurfural	Aldehyde	Malt	GC-MS	stale, vegetable oil, paper	ASBC Beer flavor database
cis-3-hexenol	Alcohol	Hops	GC-MS	green, grassy	ASBC Beer flavor database
heptyl acetate	Ester	Yeast	GC-MS	pear, fruity, sweet	ASBC Beer flavor database
hexyl acetate	Ester	Yeast	GC-MS	sweet, aromatic, perfumed	ASBC Beer flavor database
humulene epoxide II	Terpenoid	Hops	GC-MS	moldy, cedar, lime, herbal	ASBC Hop flavor database
hydrogen sulfide	Sulfur compound	Yeast	GC-FPD	eggs, rotten eggs	ASBC Beer flavor database
n-heptanol	Alcohol	Yeast	GC-MS	coconut, solvent	ASBC Beer flavor database
n-hexanol	Alcohol	Yeast	GC-MS	coconut, fruity, green	ASBC Beer flavor database The Good Scents Company
α-humulene	Terpenoid	Hops	GC-MS	balsamic, floral, grassy, herbal, spicy	ASBC Hop flavor database
β-humulene	Terpenoid	Hops	GC-MS	earthy, woody, spicy	
E-α-ionone	Terpenoid	Hops	GC-MS	floral, violet	The Good Scents Company
E-β-ionone	Terpenoid	Hops	GC-MS	raspberry, cedar, violets	ASBC Hop flavor database
Iron	Inorganic	Malt	Enzymatic assay	metallic	ASBC Beer flavor database
isoamyl acetate	Ester	Yeast	GC-FID	banana, fruity	ASBC Beer flavor database
isoamyl alcohol	Alcohol	Yeast	GC-FID	alcoholic, banana	The Good Scents Company

isoamyl lactate	Ester	Bacteria	GC-MS	fruity	The Good Scents Company
isoborneol	Terpenoid	Additive	GC-MS		
Isobutanol	Alcohol	Yeast, Aging	GC-FID	alcoholic	ASBC Beer flavor database
isobutyl acetate	Ester	Yeast	GC-FID	banana, sweet, fruity	ASBC Beer flavor database
isobutyl hexanoate	Ester	Yeast	GC-MS	fruity, pineapple	The Good Scents Company
isomaltol	Furan	Malt	GC-MS	caramel, burnt	ASBC Beer flavor database
isopentyl butanoate	Ester	Yeast	GC-MS	fruity, green, apricot	The Good Scents Company
isopentyl formate	Ester	Yeast	GC-MS	plum, solvent-like	ASBC Beer flavor database
isopentyl hexanoate	Ester	Yeast	GC-MS	fruity, banana, pineapple	ASBC Beer flavor database
isopropyl hexadecanoate	Ester	Yeast	GC-MS		
isopropyl laurate	Ester	Yeast	GC-MS		
isopropyl tetradecanoate	Ester	Yeast	GC-MS		
iso-α acids	Acid	Hops	Spectro-photometric	bitter	
kongol	Miscellaneous	Hops	GC-MS		
cis-linalool oxide	Terpenoid	Hops	GC-MS		
limonene	Terpenoid	Spices, Hops	GC-MS	citrus, orange, green, fruity	ASBC Hop flavor database
linalyl isovalerate	Terpenoid	Hops	GC-MS	herbal, citrus, bergamot, lavender	The Good Scents Company
lactic acid	Acid	Bacteria	Enzymatic assay	acidic, neutral, pleasant sour	ASBC Beer flavor database
linalool	Terpenoid	Yeast, Hops, Spices	GC-MS	floral, fruity, citrus, rosewood	ASBC Hop flavor database
13-epi-manoyl oxide	Terpenoid	Spices	GC-MS		
2E,6E-methyl farnesoate	Terpenoid	Hops	GC-MS		
2-methyl-1-butanol	Alcohol	Yeast	GC-MS	malty, alcoholic, banana	ASBC Beer flavor database
2-methyl-benzofuran	Furan	Malt	GC-MS		
2-methylbutyl octanoate	Ester	Yeast	GC-MS		
2-methyltetrahydrofuran-3-one	Ketone	Malt, Aging	GC-MS	bready, nutty	The Good Scents Company
3-methyl-2-butenal	Aldehyde	Yeast	GC-MS	fruity, nutty	The Good Scents Company
3-methyl-2-hexanol	Alcohol	Yeast	GC-MS		
3-methylbutanal	Alcohol	Malt, Aging	GC-MS	malt, cherry, almond, chocolate	ASBC Beer flavor database
4-methoxysalicylaldehyde	Aldehyde	Spices	GC-MS	vanilla	The Good Scents Company
4-methyl-1-pentanol	Alcohol	Yeast	GC-MS	nutty	The Good Scents Company
5-methylfurfural	Aldehyde	Malt, Wood	GC-MS	almond, marzipan	ASBC Beer flavor database
E-ortho-methoxy cinnamaldehyde	Aldehyde	Spices	GC-MS	spicy, cinnamon, medicinal	The Good Scents Company
manool oxide	Terpenoid	Spices	GC-MS		
manool	Terpenoid	Spices	GC-MS		

methanethiol	Sulfur compound	Malt, Yeast	GC-FPD	Putrefaction, drains, rotting vegetables	ASBC Beer flavor database
methionol	Sulfur compound	Aging	GC-MS	potato	ASBC Beer flavor database
methyl geranate	Terpenoid	Hops	GC-MS	waxy, green, fruity, floral	ASBC Hop flavor database
methyl heptenone	Ketone	Yeast	GC-MS	citrus, fruity	The Good Scents Company
methyl hexadecanoate	Ester	Yeast	GC-MS		
methyl isoeugenol	Terpenoid	Spices	GC-MS	spicy	The Good Scents Company
methyl para-anisate	Terpenoid	Spices	GC-MS		
methylpyrazine	Pyrazine	Malt	GC-MS	nutty, roasted, musty	The Good Scents Company
tau-muurolol	Terpenoid	Hops	GC-MS	spicy, herbal, honey	The Good Scents Company
x-methyl-1-octanol	Alcohol	Yeast	GC-MS		
y-methyl-1-octanol	Alcohol	Yeast	GC-MS		
α-muurolol	Terpenoid	Hops	GC-MS	herbal, honey	The Good Scents Company
2-nonanol	Alcohol	Hops	GC-MS	coconut	ASBC Beer flavor database
E-2-nonenal	Aldehyde	Malt, Aging	GC-MS	papery, cardboard, stale	ASBC Beer flavor database
E-nerolidol	Terpenoid	Hops	GC-MS	floral	The Good Scents Company
gamma-nonalactone	Ketone	Aging, Yeast	GC-MS	coconut, vanilla, glue	ASBC Beer flavor database
neo-allo-ocimene	Terpenoid	Hops	GC-MS		
nerol oxide	Terpenoid	Hops	GC-MS	green, herbal, narcissus	The Good Scents Company
nerol	Terpenoid	Hops	GC-MS	floral, citrus, roses	ASBC Beer flavor database
neryl formate	Terpenoid	Hops	GC-MS	floral, rose, herbal	The Good Scents Company
neryl isobutanoate	Terpenoid	Hops	GC-MS	fruity, raspberry, strawberry	The Good Scents Company
neryl isovalerate	Terpenoid	Hops	GC-MS	floral, citrus, sage, bergamot	The Good Scents Company
neryl propanoate	Terpenoid	Hops	GC-MS	fruity, berry, jam, floral	The Good Scents Company
n-nonanal	Aldehyde	Yeast	GC-MS	bitter, astringent, cardboard	ASBC Beer flavor database
n-nonanol	Alcohol	Yeast	GC-MS	coconut, walnut, oily	ASBC Beer flavor database
Z-nerolidol	Terpenoid	Hops	GC-MS	floral	The Good Scents Company
1-octen-3-ol	Alcohol	Yeast	GC-MS	green, earthy, mushroom, metallic	ASBC Beer flavor database The Good Scents Company
E-β-ocimene	Terpenoid	Hops	GC-MS	citrus, terpene	ASBC Hop flavor database
n-octanol	Alcohol	Yeast	GC-MS	coconut, walnut, oily	ASBC Beer flavor database
ocimenone	Terpenoid	Hops	GC-MS		
octyl acetate	Ester	Yeast	GC-FID	coconut, vegetable oil, aromatic	ASBC Beer flavor database
1-propanol	Alcohol	Yeast		alcoholic, solvent-like	ASBC Beer flavor database
2-pentyl furan	Furan	Malt	GC-MS	fruity, green, metallic	The Good Scents Company
2-phenyl ethyl butyrate	Ester	Yeast	GC-MS	floral, musty	The Good Scents Company

n-pentanol	Alcohol	Yeast	GC-MS	sweet, aromatic, perfumed	ASBC Beer flavor database
n-propyl acetate	Ester	Yeast	GC-MS	solvent, sweet, perfumed	ASBC Beer flavor database
pentyl octanoate	Ester	Yeast	GC-MS	earthy, wine	The Good Scents Company
perillyl acetate	Ester	Spices	GC-MS	fruiy, geranium, herbal, spicy, floral	The Good Scents Company
pH	Miscellaneous		Colorimetric		
phenethyl acetate	Ester	Yeast	GC-FID	roses, honey, fruity, floral	ASBC Beer flavor database
phenethyl alcohol	Alcohol	Yeast	GC-FID	floral, roses	ASBC Beer flavor database
phenylacetaldehyde	Aldehyde	Yeast, Aging	GC-MS	floral, honey	ASBC Beer flavor database
prenyl isobutyrate	Ester	Yeast	GC-MS	fruity, berry	The Good Scents Company
propylene glycol	Miscellaneous	Additive	GC-MS		
protein	Miscellaneous	Malt, Yeast	Enzymatic assay		
α-phellandrene	Terpenoid	Hops, Spices	GC-MS	terpentine, pepper, citrus	The Good Scents Company
β-phellandrene	Terpenoid	Hops	GC-MS	pine, terpentine, fruity, spicy	ASBC Hop flavor database
spathulenol	Terpenoid	Spices	GC-MS	herbal, earthy, fruity	The Good Scents Company
styrene	Miscellaneous	Yeast	GC-MS	balsamic, sweet, plastic	The Good Scents Company
sugars	Carbohydrate	Malt	Enzymatic assay	sweet	
sulfur dioxide	Sulfur compound	Yeast	Enzymatic assay	sulfytic, burnt matchsticks	ASBC Beer flavor database
sum of acids			-		
sum of esters			-		
sum of hop aroma			-		
sum of sulfur compounds			-		
1-α-terpineol	Terpenoid	Hops, Yeast	GC-MS	pine, floral	ASBC Hop flavor database
cis-β-terpineol	Terpenoid	Hops	GC-MS		
n-tetradecanol	Alcohol	Yeast	GC-MS	coconut, fruity, waxy	The Good Scents Company
terpinen-4-ol acetate	Terpenoid	Hops	GC-MS		
terpinen-4-ol	Terpenoid	Spices	GC-MS	woody, ceding, menthlic, citrus	ASBC Hop flavor database
terpinolene	Terpenoid	Spices	GC-MS	woody, fruity, piney	ASBC Hop flavor database
terpiryl acetate	Terpenoid	Hops	GC-MS		
α-terpinyl isobutanoate	Terpenoid	Hops	GC-MS		
2-undecanol	Alcohol	Hops	GC-MS	perfumed, sweet, coconut, varnish, musty	ASBC Beer flavor database
2-undecanone	Ketone	Hops, Yeast	GC-MS	varnish, bitter, green, geranium	ASBC Beer flavor database
unfermentables	Miscellaneous	Malt	Densimetric		
4-vinylguaiacol	Phenol	Yeast, Wood	GC-MS	clove, smoky, spicy, phenolic, vanilla	ASBC Beer flavor database

valencene	Terpenoid	Spices	GC-MS	citrus, green oil, wood	The Good Scents Company
vanillin	Phenol	Yeast, Spices, Wood	GC-MS	vanilla, sweet	ASBC Beer flavor database
vinyl caprylate	Ester	Yeast	GC-MS		

Supplementary Table S2: Two-sided ANOVA p-values for samples repeated across multiple tasting sessions. Columns indicate beer samples that were repeated at least once, rows correspond to sensory aspects. A p-value smaller than 0.05 was observed for 25 out of the 600 tests.

Attribute	Beer 23	Beer 37	Beer 75	Beer 114	Beer 127	Beer 139	Beer 185	Beer 202	Beer 214	Beer 219	Beer 224	Beer 238
Malt aroma - overall	0.83	0.07	0.38	0.87	0.18	0.88	0.28	0.05	0.54	1.00	0.38	0.78
Malt aroma - grain	0.54	0.83	0.71	0.15	0.13	0.45	0.21	0.65	0.80	0.41	0.92	0.59
Malt aroma - bread	0.23	0.23	0.33	0.76	0.40	0.27	1.00	0.08	1.00	0.81	0.78	0.74
Malt aroma - caramel	1.00	0.02	0.33	1.00	1.00	0.87	1.00	0.09	0.62	0.34	0.37	0.39
Malt aroma - smoked	0.33	0.07	0.66	0.16	0.66	0.33	1.00	0.25	1.00	1.00	0.50	0.52
Hops aroma - overall	0.75	0.20	0.85	0.90	0.27	0.14	0.84	0.26	0.31	0.01	0.23	0.71
Hops aroma - citrus	0.88	0.59	0.66	0.58	0.26	0.30	0.48	0.26	1.00	0.13	0.15	0.48
Hops aroma - tropical fruit	0.33	0.79	1.00	1.00	0.06	0.33	0.34	1.00	1.00	0.00	0.95	0.24
Hops aroma - grassy	0.63	0.19	0.53	0.45	0.00	0.20	0.14	0.45	0.54	0.18	0.62	0.22
Hops aroma - woody	0.33	0.18	0.33	0.39	0.50	1.00	1.00	0.49	1.00	0.59	0.34	0.71
Esters aroma - overall	0.40	0.71	0.85	0.30	0.72	0.81	0.06	0.81	0.55	0.01	0.00	0.19
Esters aroma - nail polish	0.56	0.34	0.76	0.52	0.69	0.73	0.10	0.24	0.54	0.00	0.38	0.06
Esters aroma - banana	0.79	0.87	0.79	0.69	0.61	0.50	0.73	1.00	0.33	0.85	0.00	0.44
Esters aroma - floral	0.66	0.33	0.33	0.91	0.39	1.00	0.34	0.49	0.31	0.59	0.81	0.48
Esters aroma - fruity	0.59	0.09	0.57	0.93	0.42	0.33	0.34	0.59	1.00	0.17	0.45	0.54
Malt taste - overall	1.00	0.54	0.83	0.49	0.64	0.60	0.71	0.13	0.86	0.11	0.15	1.00
Malt taste - grain	0.46	0.82	0.86	0.14	0.46	0.27	0.60	0.80	0.68	0.64	0.06	0.52
Malt taste - bread	1.00	0.58	0.28	0.25	0.12	0.25	1.00	0.50	0.18	0.14	0.58	0.83
Malt taste - caramel	0.33	0.83	1.00	0.32	0.99	0.30	0.34	0.46	1.00	0.57	0.17	0.48
Malt taste - smoked	0.33	0.91	1.00	0.84	0.57	0.12	0.34	0.54	0.18	0.55	0.70	0.48
Hops taste - overall	0.06	0.11	0.59	0.57	0.03	0.78	0.62	0.18	0.56	0.07	0.90	0.85
Hops taste - citrus	0.66	0.56	0.79	0.60	0.42	0.41	1.00	1.00	0.68	0.15	0.56	0.46
Hops taste - tropical fruit	1.00	0.33	1.00	0.67	0.42	1.00	1.00	1.00	1.00	0.00	0.95	0.66
Hops taste - grassy	0.17	0.44	1.00	0.29	0.00	0.66	0.83	0.10	0.38	0.30	0.54	0.11
Hops taste - woody	0.33	0.85	0.16	0.73	0.39	0.33	0.79	0.89	1.00	1.00	0.34	0.32
Esters taste - overall	0.43	0.50	0.73	0.32	0.07	0.51	0.56	0.79	0.25	0.10	0.15	0.64
Esters taste - nail polish	0.74	0.23	0.76	0.22	0.15	0.87	0.34	0.42	0.54	0.01	0.52	0.72
Esters taste - banana	0.18	0.22	0.84	0.50	0.33	0.85	0.34	1.00	0.54	0.74	0.06	0.59
Esters taste - floral	0.81	0.16	0.33	0.88	0.42	1.00	0.34	1.00	0.49	0.64	0.93	1.00
Esters taste - fruity	0.59	0.05	0.48	0.89	0.31	0.54	0.34	1.00	0.18	0.34	0.48	0.33
Body fullness	0.79	0.05	0.81	0.44	0.01	0.61	0.80	1.00	0.71	0.21	0.62	0.71
Bitterness	0.16	0.61	0.85	1.00	0.44	0.76	0.86	0.77	0.38	0.48	0.51	0.33
Carbonation	0.29	0.54	0.43	0.16	0.85	0.31	1.00	0.87	0.46	0.21	0.68	0.41
Aftertaste	0.66	0.47	0.40	0.44	0.86	0.51	0.44	1.00	0.01	0.26	0.92	0.22
Alcohol	0.56	1.00	0.75	0.12	0.03	0.66	0.80	1.00	1.00	0.00	0.62	0.56
Sweetness	0.55	0.86	0.20	0.37	0.24	0.17	1.00	0.57	0.21	0.72	0.77	0.82
Acidity	1.00	0.77	0.87	0.79	0.00	0.20	0.36	0.73	1.00	0.51	0.75	0.00
Dimethyl sulfide	0.45	1.00	0.59	1.00	0.01	0.85	0.32	0.30	1.00	0.80	0.23	0.46

Metallic	0.41	0.10	0.22	0.18	0.07	0.88	0.02	0.58	1.00	0.17	0.12	0.75
4-vinyl guaiacol	0.32	0.48	0.33	0.36	0.16	0.41	0.34	0.33	1.00	0.69	0.08	0.28
Trans-2-nonenal	0.82	0.39	0.11	0.11	0.82	0.20	0.50	0.68	1.00	0.40	0.76	0.66
Diacetyl	0.42	0.02	0.33	0.32	1.00	0.93	1.00	0.35	1.00	0.61	0.04	0.62
Stale hops	0.33	0.04	0.46	0.22	0.33	0.33	0.15	0.46	1.00	0.09	1.00	1.00
Orange	0.33	1.00	1.00	0.16	0.33	0.41	1.00	1.00	1.00	0.34	0.09	1.00
Coriander	1.00	1.00	0.33	1.00	1.00	1.00	1.00	0.49	1.00	1.00	0.08	1.00
Clove	0.18	1.00	1.00	1.00	0.42	0.41	1.00	0.49	0.03	1.00	0.50	0.84
Lactic acid	1.00	1.00	0.71	1.00	1.00	1.00	1.00	1.00	1.00	1.00	1.00	0.33
Acetic acid	1.00	1.00	0.19	1.00	1.00	1.00	1.00	1.00	1.00	1.00	1.00	1.00
Barnyard	0.33	0.04	0.46	0.22	0.66	0.33	0.15	0.46	1.00	0.09	1.00	1.00
Overall appreciation	1.00	0.30	0.90	0.52	0.09	0.28	0.22	0.43	0.63	0.85	0.33	0.54

Supplementary Table S3: Comparison between different models trained to predict tasting panel descriptors.
Performance metric is the Coefficient of Determination between predictions and observations.

Attribute	ABR	ANN	ET	GBR	Lasso	LR	PLSR	RF	SVR	XGBR
Malt aroma - overall	0.41	0.29	0.38	0.37	0.35	-2.21	-4.46	0.4	-7.65	0.3
Malt aroma - grain	-0.12	-0.23	0.12	0.01	0.02	-1.11	-1.85	0.04	0.04	-0.24
Malt aroma - bread	0.11	0.04	0.07	0.01	0.08	-13.44	-3.25	0.08	-0.28	0.06
Malt aroma - caramel	0.29	0.1	0.36	0.25	0.24	-3.8	-3.68	0.35	-0.04	0.35
Malt aroma - smoked	0.23	-0.09	0.06	0.11	-0.05	-1	-2.14	0.04	-0.03	0.23
Hops aroma - overall	0.11	0.31	0.2	0.16	0.08	-1.51	-0.08	0.11	0.18	-0.05
Hops aroma - citrus	-0.01	-0.08	0.05	-0.02	0	-0.18	-2.91	0.03	0	0.05
Hops aroma - tropical fruit	-0.2	-0.02	-0.27	-0.12	-0.07	-0.88	-1.23	-0.42	-0.16	-0.37
Hops aroma - grassy	0.22	0.07	0.22	0.16	0.02	-1.79	-4.11	0.23	-9.16	0.22
Hops aroma - woody	0.06	-0.12	0.09	0.1	-0.03	-0.9	-1.96	0.09	-6.32	0.02
Esters aroma - overall	0.16	-0.14	0.11	0.11	-0.55	-10.96	-5.61	0.15	-1.28	0.09
Esters aroma - nail polish	-0.01	-0.33	0.06	-0.05	0.06	-14.57	-0.05	0.08	0.09	0.03
Esters aroma - banana	0.07	-0.11	0.13	0.17	-0.1	-2.01	-0.99	0.13	-2610.61	0.19
Esters aroma - floral	0.03	-0.08	0	0	-1.38	-0.73	-7.16	0.02	0.01	0.02
Esters aroma - fruity	0.11	-2	0.08	0.09	0.02	0.06	0.25	0.02	-0.15	0.05
Malt taste - overall	0.42	-0.18	0.41	0.4	-0.88	-2.77	-6.58	0.42	-31.11	0.39
Malt taste - grain	-0.03	-0.23	0.04	-0.02	-0.04	-1.79	-1.13	0.09	-535.79	-0.07
Malt taste - bread	0.21	-0.07	0.17	0.17	-0.76	-2.1	-6.04	0.16	-5.99	0.07
Malt taste - caramel	0.17	-0.26	0.29	0.28	0	-1.21	-6.21	0.3	0.28	0.23
Malt taste - smoked	0.42	0.36	0.31	0.3	0.44	-5.63	-2.84	0.35	0.19	0.37
Hops taste - overall	0.29	-0.09	0.37	0.39	0.29	-9.02	-1.59	0.36	0.33	0.31
Hops taste - citrus	0.04	-0.28	0.01	0.05	0.01	-0.27	-0.18	0	-0.08	-0.05

Hops taste - tropical fruit	-0.14	-0.01	-0.17	-0.26	-0.04	-0.41	-2.39	-0.18	-0.06	-0.27
Hops taste - grassy	0.28	-0.34	0.3	0.28	0.23	-5.91	-1.08	0.31	0.29	0.36
Hops taste - woody	0.12	0.15	0.14	0.16	0.04	-3.8	-2.02	0.13	-1.48	0.07
Esters taste - overall	0.31	-0.03	0.27	0.27	0.1	-9.24	0.35	0.29	0.29	0.23
Esters taste - nail polish	0.18	-0.12	0.2	0.14	0.06	-15.81	-2.66	0.19	-5.04	0.23
Esters taste - banana	0.18	-1.25	0.16	0.1	-0.02	-3.01	-0.87	0.17	0.12	0.08
Esters taste - floral	0	-0.47	-0.01	-0.03	0	-1.98	-4.38	0	-4656.64	-0.07
Esters taste - fruity	0.15	0.09	0.14	0.15	-0.11	-0.02	-0.04	0.17	-0.47	0.18
Acidity	0.73	0.62	0.71	0.75	0.63	0.46	-6.5	0.74	-8.92	0.73
Bitterness	0.64	0.23	0.59	0.64	0.55	-10.29	-0.76	0.62	0.56	0.66
Sweetness	0.46	0.1	0.39	0.45	0.15	-0.71	0.36	0.31	-3724455	0.36
4-vinyl guaiacol	0.32	0.11	0.21	0	0.05	-2.32	-8.88	0.32	0.24	0.26
Diacetyl	-0.12	-0.01	-0.05	-0.17	-0.08	-3.71	-5.84	-0.13	-0.13	-0.17
Dimethyl sulfide	0.05	-0.01	0.01	0.01	-0.01	-3.07	-43.67	0.08	-19100.4	0.02
Metallic	0.08	-0.04	0.07	0.14	0	-1.18	-21.73	0.12	0.17	0.07
Stale hops	0.04	-0.2	0.01	0.11	0	-5.53	-4.33	0.05	0.04	-0.04
Trans-2-nonenal	-0.05	-0.02	0.03	-0.05	-0.07	-1.74	-0.73	-0.01	-1249.57	-0.26
Orange	0.26	-0.13	0.2	0.27	-0.11	-0.42	0.16	0.23	-564.4	-0.04
Coriander	0.26	-0.21	0.11	0.07	0.25	0.18	0.2	0.12	-605474	0.19
Clove	-0.14	-0.01	0.02	0.23	0	0.03	-8.88	0.03	-5.34	-0.16
Lactic acid	0.63	0.52	0.6	0.69	0.58	-0.41	-6.96	0.65	-1.47	0.58
Acetic acid	0.65	0.38	0.67	0.58	0.5	-1.86	-23.69	0.66	-103282	0.7
Barnyard	0.04	-0.07	0.02	0.04	-0.01	-6.2	-5.63	0.01	0	-0.08
Alcohol	0.71	0.53	0.75	0.74	0.46	-14.44	-1.84	0.77	-7.36	0.75
Aftertaste	0.45	0.52	0.43	0.4	0.44	-10.9	0.28	0.43	-6.06	0.44
Body	0.55	0.22	0.52	0.54	0.45	-8.21	-1.15	0.53	-2.43	0.45
Carbonation	0.31	-0.14	0.36	0.21	0.23	-13.83	-11.22	0.34	-30.51	0.29
Overall appreciation	0.06	0.06	0.1	0.09	0	-4.57	-26.93	0.07	-10.68	0.05

Supplementary Table S4: Comparison between different models trained to predict RateBeer review scores. The performance metric is the Pearson correlation between predictions and observations.

Attribute	ABR	ANN	ET	GBR	Lasso	LR	PLSR	RF	SVR	XGBR
Ratebeer score	0.6	0.7	0.62	0.67	0.62	-11.41	0.62	0.62	0.58	0.65
Aroma appreciation	0.64	0.69	0.64	0.71	0.63	-9.19	0.63	0.65	0.64	0.68
Appearance	0.59	0.61	0.63	0.68	0.61	-10.3	0.03	0.62	0.61	0.66
Taste appreciation	0.6	0.62	0.61	0.71	0.66	-11.69	0.63	0.64	0.58	0.67
Palate	0.6	0.51	0.62	0.64	0.63	-12.37	0.36	0.65	-4.42	0.65
Overall appreciation	0.6	0.61	0.6	0.67	0.6	-11.29	0.62	0.61	0.56	0.69

Supplementary Table S5: Feature importance ranks of beer styles in a model trained with style identifiers. Most of the styles are ranked as less important than all chemical compounds (rank 241-253) for predicting RateBeer appreciation. Only two styles (Low/No alcohol and Strong ale) contribute noticeably to the model's decisions.

Beer style	MDI importance rank	SHAP importance rank
Low/No alcohol	11	12
Strong ale	28	17
Blond	173	162
Wheat	206	232
Dubbel	233	236
Flanders old brown	236	229
Faro	237	240
Saison	239	235
Pils/Lager	240	239
Tripel	241	241
Amber	242	243
Hoppy	243	242
Stout/Porter	244	244
West Flanders ale	245	245
Kriek	246	247
Brown	247	246
Fruitbeer	248	248
Lambic	249	249
Brut	250	250
Brett/cofermented	251	251
Christmas	252	252
Scotch	253	253

Supplementary Table S6: Average RateBeer appreciation scores per beer style. The two most important styles according the model (Supplementary Table S5), correspond to the least and most and least appreciated styles, strong ale and low/no alcohol respectively. Please note that our RateBeer scores are negative on average, because each reviewers scores were scaled to all the beers rated by that reviewer. This includes many beers outside of this dataset, which can result in a negative score when averaging over the beers that are included in the study.

**Beer style**	**Average RateBeer appreciation**
Low/No alcohol	-2.65
Pils/Lager	-1.68
Fruitbeer	-0.70
Faro	-0.68
Wheat	-0.57
Scotch	-0.44
Amber	-0.37
Blond	-0.35
Flanders old brown	-0.23
Dubbel	-0.17
Hoppy	-0.16
Tripel	-0.15
Stout/Porter	-0.14
West Flanders ale	-0.13
Brut	-0.11
Lambic	-0.09
Saison	-0.08
Christmas	-0.004
Kriek	0.04
Brett/cofermented	0.10
Brown	0.10
Strong ale	0.25

Supplementary Table S7: **Calibration standards.**

Compound	CAS-number	Supplier	Reference number
(E)-2-nonenal	18829-56-6	Merck KGaA	W321303
(E)-beta-damascenone	23726-91-2	Merck KGaA	W324300-SAMPLE-K
(E)-beta-ocimene	13877-91-3	Merck KGaA	W353901-SAMPLE
(E,E)-2,4-decadienal	25152-84-5	Merck KGaA	W313505-SAMPLE-K
1-alpha-terpineol	98-55-5	Merck KGaA	04899-250MG
1-propanol	71-23-8	Merck KGaA	96566-5ML-F
1-undecanol	112-42-5	Merck KGaA	U1001-100g
2,3-butanedione	431-03-8	Merck KGaA	11038-1ML-F
2,3-pentanedione	600-14-6	Merck KGaA	W284114-SAMPLE
2-ethyl-3-methylpyrazine	15707-23-0	Merck KGaA	W315508-SAMPLE-K
2-methylpropanal	78-83-1	Merck KGaA	82059-1ML-F
2-methyltetrahydrofuran-3-one	3188-00-9	Merck KGaA	277487-5G
2-phenethyl acetate	103-45-7	Merck KGaA	73747-1ML
3-methylbutanal	590-86-3	Merck KGaA	W269204-SAMPLE-K
3Z-hexenol	928-96-1	Merck KGaA	W256323-100G-K
5-methylfurfural	620-02-0	Merck KGaA	W270202-SAMPLE-K
acetaldehyde	75-07-0	Merck KGaA	0070-100ml
acetaldehyde	75-07-0	Merck KGaA	0070-100ml
acetic acid	64-19-7	VWR International	20104334
acetoin	513-86-0	Merck KGaA	W200832-100G-K
alpha-humulene	6753-98-6	Merck KGaA	12448-250MG
ammonia	7664-41-7	Thermo Fisher Scientific Inc.	984720
amyl acetate	628-63-7	Merck KGaA	66962-1ML
benzaldehyde	100-52-7	Merck KGaA	09143-5ML-F
benzyl alcohol	100-51-6	Merck KGaA	08421-5ML-F
betaglucan	-	Thermo Fisher Scientific Inc.	984383
Iso-alpha acids (bitterness)	25522-96-7	Labor Veritas AG	DCHA-ISO ICS-I3
butanol	71-36-3	Merck KGaA	19422-5ML
carbon disulfide	75-15-0	Merck KGaA	335266
citronellol	106-22-9	Merck KGaA	C83201-5G
diethyl succinate	123-25-1	Merck KGaA	W237701-SAMPLE-K
diethyl sulfide	352-932	Merck KGaA	107247-5ml
dimethyl sulfide	75-18-3	Merck KGaA	W274615-SAMPLE-K
E-caryophyllene	87-44-5	Merck KGaA	W225207-SAMPLE-K
ethanol	64-17-5	VWR International	20821321
ethyl 2.methyl butyrate	7452-79-1	Merck KGaA	306886-5ml
ethyl acetate	141-78-6	Merck KGaA	58958-5ML
ethyl butyrate	123-66-0	Merck KGaA	75563-1ml
ethyl decanoate	110-38-3	Merck KGaA	00733-1ml
ethyl hexanoate	123-66-0	Merck KGaA	08375-1ml

ethyl isobutyrate	97-62-1	Merck KGaA	19536-1ml
ethyl isovalerate	108-64-5	Merck KGaA	71607-1ML
ethyl lactate	687-47-8	Merck KGaA	69799-250ml
ethyl nicotinate	614-18-6	Merck KGaA	E-406609-100ml
ethyl octanoate	106-32-1	Merck KGaA	44879-1ML
ethyl pentanoate	539-82-2	Merck KGaA	30784-1ml
ethyl propanoate	105-37-3	Merck KGaA	96727-1ML
eugenol	97-53-0	Merck KGaA	E51791-5G
fructose	57-48-7	Thermo Fisher Scientific Inc.	984380
furfural	98-01-1	Merck KGaA	185914
furfuryl alcohol	98-00-0	Merck KGaA	185930-50G
gamma-nonalactone	104-61-0	Merck KGaA	W278106-SAMPLE-K
geraniol	106-24-1	Merck KGaA	W250708-SAMPLE-K
glucose	50-99-7	Thermo Fisher Scientific Inc.	984380
glycerol	56-81-5	Thermo Fisher Scientific Inc.	984386
hexyl acetate	142-92-7	Merck KGaA	25539-1ML
hydrogen sulfide	207683-19-0	Merck KGaA	161527-100G
iron	7782-63-0	Merck KGaA	F7002-250G
isoamyl alcohol	123-51-3	Merck KGaA	309435
isobutanol	78-83-1	Merck KGaA	82059-1ML-F
isobutyl acetate	110-19-0	Merck KGaA	94823
isopentyl acetate	123-92-2	Merck KGaA	123-92-2
isovaleraldehyde	590-86-3	Merck KGaA	W269204-SAMPLE-K
lactic acid	50-21-5	Thermo Fisher Scientific Inc.	984382
linalool	126-91-0	Merck KGaA	62139-25ML
methanethiol	5188-07-08	Merck KGaA	281018-5G
methylpyrazine	109-08-0	Merck KGaA	M75608-25G
naphtalene	91-20-3	Merck KGaA	84679-1G
n-hexanol	111-27-3	Merck KGaA	73117-1ML-F
octyl acetate	112-14-1	Merck KGaA	04622-1ML
para-vinyl-guaiacol	7786-61-0	Merck KGaA	w267511-sample-K
pH	-	Thermo Fisher Scientific Inc.	984331; 984332
phenethyl acetate	103-45-7	Merck KGaA	73747-1ML
phenethyl alcohol	60-12-8	Merck KGaA	PHR1122-1.5G
phenetyl acetate	103-45-7	Merck KGaA	73747-1ML
phenylacetaldehyde	122-78-1	Merck KGaA	W287407-SAMPLE-K
propyl acetate	109-60-4	Merck KGaA	40858
protein	-	Merck KGaA	A9418
styrene	100-42-5	Merck KGaA	100-42-5
sucrose	7681-57-4	Thermo Fisher Scientific Inc.	984380
sulfite	7681-57-4	Merck KGaA	S9000-500G
Z-nerolidol	7212-44-4	Merck KGaA	W277207

Supplementary Table S8: List of software packages, with their corresponding version and citation.

Software	Packages	Version	Reference
Python 3.6	langdetect	1.0.7	Nakatani, S. Langdetect: Language Detection Library for Java. (2010).[3]
Python 3.6	langid	1.1.6	Lui, M. & Baldwin, T. langid.py: An Off-the-shelf Language Identification Tool. in Proceedings of the ACL 2012 System Demonstrations 25-30, (2012).[4]
Python 3.6	nltk	3.2.5	Bird, S., Klein, E. & Loper, E. Natural language processing with Python: analyzing text with the natural language toolkit. (2009).[5]
Python 3.6	scrapy	1.5.1	Kouzis-Loukas, D. Learning Scrapy. (2016).[6]
Python 3.9.16	anaconda	2023.07	Anaconda Software Distribution. Anaconda Documentation. (2023).[7]
Python 3.9.16	conda	23.5.0	Anaconda Software Distribution. Anaconda Documentation. (2023).[7]
Python 3.9.16	joblib	1.2.0	Joblib Development Team. Joblib: running Python functions as pipeline jobs. (2020).[8]
Python 3.9.16	numpy	1.24.3	Harris, C. R. et al. Array programming with NumPy. Nature 585, 357–362 (2020).[9]
Python 3.9.16	pandas	1.5.3	McKinney, W. & al. Data structures for statistical computing in python. in Proceedings of the 9th Python in Science Conference vol. 445 51–56 (2010).[10]
Python 3.9.16	scikit-learn	1.2.2	Pedregosa, F. et al. Scikit-learn: Machine learning in Python. J. Mach. Learn. Res. 12, 2825–2830 (2011).[11]
Python 3.9.16	seaborn	0.12.2	Waskom, M. et al. Seaborn. vol. 0 (2017).[12]
Python 3.9.16	shap	0.41.0	Lundberg, S. M., Erion, G. & Chen, H. From local explanations to global understanding with explainable AI for trees. Nat. Mach. Intell. 2, 56–67 (2020).[13]
Python 3.9.16	xgboost	1.7.3	Chen, T. & Guestrin, C. XGBoost: A Scalable Tree Boosting System. in Proceedings of the 22nd ACM SIGKDD International Conference on Knowledge Discovery and Data Mining 785–794 (2016). doi:10.1145/2939672.2939785.[14]
R 4.1.3	abind	1.4.5	Plate, T. abind: Combine Multidimensional Arrays. (2003).[15]
R 4.1.3	baseline	1.3.1	Liland, K. H. baseline: Baseline Correction of Spectra. (2011).[16]
R 4.1.3	broom	0.7.12	Couch, S. broom: Convert Statistical Objects into Tidy Tibbles. (2014).[17]
R 4.1.3	Cairo	1.5.15	Urbanek, S. Cairo: R Graphics Device using Cairo Graphics Library for Creating High-Quality. (2007).[18]
R 4.1.3	car	3.0.12	Fox, J. car: Companion to Applied Regression. (2001).[19]
R 4.1.3	caTools	1.18.2	Dietze, M. caTools: Tools: Moving Window Statistics, GIF, Base64, ROC AUC, etc. (2005).[20]
R 4.1.3	clValid	0.7	Brock, G., Pihur, V., Datta, S. & Datta, S. clValid: An R Package for Cluster Validation. J. Stat. Softw. 25, 1–22 (2008).[21]

R 4.1.3	cobs	1.3.4	Ng, P. & Maechler, M. A Fast and Efficient Implementation of Qualitatively Constrained Quantile Smoothing Splines. Stat. Model. 7, 315–328 (2007).[22]
R 4.1.3	colorRamps	2.3	Keitt, T. colorRamps: Builds Color Tables. R package version 2.3. (2007).[23]
R 4.1.3	data.table	1.14.2	Dowle, M. data.table: Extension of `data.frame`. R package version 1.14.2. (2006).[24]
R 4.1.3	devtools	2.4.3	Wickham, H., Hester, J., Chang, W. & Bryan, J. devtools: Tools to Make Developing R Packages Easier. (2022).[25]
R 4.1.3	doParallel	1.0.17	Daniel, F. doParallel: Foreach Parallel Adaptor for the 'parallel' Package. (2011).[26]
R 4.1.3	doSNOW	1.0.20	Daniel, F. doSNOW: Foreach Parellel Adaptor for the 'snow' Package. (2022).[27]
R 4.1.3	dplyr	1.0.8	Wickham, H., François, R., Henry, L., Müller, K. & Vaughan, D. dplyr: A Grammar of Data Manipulation. (2023).[28]
R 4.1.3	export	0.3.0	Wenseleers, T. export: Streamlined Export of Graphs and Data Tables. (2018).[29]
R 4.1.3	fingerprint	3.5.7	Guha, R. fingerprint: Functions to Operate on Binary Fingerprint Data. (2006).[30]
R 4.1.3	flexmix	2.3.17	Gruen, B. flexmix: Flexible Mixture Modeling. (2003).[31]
R 4.1.3	flextable	0.7.0	Gohel, D. flextable: Functions for Tabular Reporting. (2017).[32]
R 4.1.3	fpc	2.2.9	Hennig, C. fpc: Flexible Procedures for Clustering. (2003).[33]
R 4.1.3	ggplot2	3.3.5	Wickham, H. ggplot2: Elegant Graphics for Data Analysis. (Springer-Verlag New York, 2016).[34]
R 4.1.3	glmnet	4.1.3	Friedman, J., Hastie, T. & Tibshirani, R. Regularization Paths for Generalized Linear Models via Coordinate Descent. J. Stat. Softw. 33, 1–22 (2010).[35]
R 4.1.3	grDevices	4.1.3	R Core Team. R: A language and environment for statistical computing. (2022).[36]
R 4.1.3	gWidgets	0.0.54.2	Verzani, J. gWidgets: gWidgets API for building toolkit-independent, interactive GUIs. (2014).[37]
R 4.1.3	Hmisc	4.6.0	Harrell, F. E., Jr. Hmisc: Harrell Miscellaneous. (2003).[38]
R 4.1.3	httr	1.4.2	Wickham, H. httr: Tools for Working with URLs and HTTP. (2023).[39]
R 4.1.3	MASS	7.3.55	Venables, W. N. & Ripley, B. D. Modern Applied Statistics with S. (2002).[40]
R 4.1.3	Matrix	1.4.0	Maechler, M. Matrix: Sparse and Dense Matrix Classes and Methods. (2000).[41]
R 4.1.3	MESS	0.5.7	Ekstrm, C. T. MESS: Miscellaneous Esoteric Statistical Scripts. (2012).[42]
R 4.1.3	metaMS	1.30.0	Wehrens, R., Weingart, G. & Mattivi, F. metaMS: An open-source pipeline for GC-MS-based untargeted metabolomics. J Chrom B 966, 109–116 (2014).[43]
R 4.1.3	miscTools	0.6.26	Henningsen, A. miscTools: Miscellaneous Tools and Utilities. (2009).[44]

R 4.1.3	mvoutlier	2.1.1	Filzmoser, P. mvoutlier: Multivariate Outlier Detection Based on Robust Methods. (2004).[45]
R 4.1.3	mzR	2.28.0	Chambers, C. M. et al. A cross-platform toolkit for mass spectrometry and proteomics. Nat Biotech 30, 918–920 (2012).[46]
R 4.1.3	ncdf4	1.19	Pierce, D. ncdf4: Interface to Unidata netCDF (Version 4 or Earlier. (2010).[47]
R 4.1.3	nnet	7.3.17	Ripley, B. nnet: Feed-Forward Neural Networks and Multinomial Log-Linear Models. (2009).[48]
R 4.1.3	nnls	1.4	Mullen, K. nnls: The Lawson-Hanson algorithm for non-negative least squares(NNLS. (2007).[49]
R 4.1.3	officer	0.4.2	Gohel, D. officer: Manipulation of Microsoft Word and PowerPoint Documents. (2023).[50]
R 4.1.3	openxlsx	4.2.5	Schauberger, P. & Walker, A. openxlsx: Read, Write and Edit xlsx Files. (2022).[51]
R 4.1.3	OrgMassSpecR	0.5.3	Dodder, N. OrgMassSpecR: Organic Mass Spectrometry. (2010).[52]
R 4.1.3	pbapply	1.5.0	Solymos, P. pbapply: Adding Progress Bar to '*apply' Functions. (2010).[53]
R 4.1.3	pheatmap	1.0.12	Kolde, R. pheatmap: Pretty Heatmaps. (2010).[54]
R 4.1.3	plyr	1.8.7	Wickham, H. The Split-Apply-Combine Strategy for Data Analysis. J. Stat. Softw. 40, 1–29 (2011).[55]
R 4.1.3	proxy	0.4.26	Meyer, D. proxy: Distance and Similarity Measures. (2007).[56]
R 4.1.3	ptw	1.9.16	Wehrens, R. ptw: Parametric Time Warping. (2009).[57]
R 4.1.3	quantreg	5.88	Koenker, R. quantreg: Quantile Regression. (2009).[58]
R 4.1.3	R.utils	2.11.0	Bengtsson, H. R.utils: Various Programming Utilities. (2005).[59]
R 4.1.3	Rcpp	1.0.8.3	Eddelbuettel, D. Rcpp: Seamless R and C++ Integration. R package version 1.0.8.3. (2008).[60]
R 4.1.3	RCurl	1.98.1.6	C.R.A.N. Team. RCurl: General Network (HTTP/FTP/...) Client Interface for R. (2004).[61]
R 4.1.3	reshape	0.8.8	Wickham, H. reshape: Flexibly Reshape Data. (2005).[62]
R 4.1.3	rgl	0.108.3	Murdoch, D. rgl: 3D Visualization Using OpenGL. R package version 0.108.3. (2004).[63]
R 4.1.3	RGtk2	2.20.36.2	Lawrence, M. RGtk2: R Bindings for Gtk 2.8.0 and Above. (2018).[64]
R 4.1.3	rJava	1.0.6	Urbanek, S. rJava: Low-Level R to Java Interface. (2005).[65]
R 4.1.3	rjson	0.2.21	Couture-Beil, A. rjson: JSON for R. (2007).[66]
R 4.1.3	RMassBank	3.4.0	Stravs, M. A., Schymanski, E. L., Singer, H. & Hollender, J. Automatic Recalibration and Processing of Tandem Mass Spectra using Formula Annotation. J. Mass Spectrom. 48, 188 (2013).[67]
R 4.1.3	rvg	0.2.5	Gohel, D. rvg: R Graphics Devices for Vector Graphics Output. (2016).[68]
R 4.1.3	signal	0.7.7	Ligges, U. signal: Signal Processing. (2006).[69]
R 4.1.3	snow	0.4.4	Tierney, L. snow: Simple Network of Workstations. (2003).[70]
R 4.1.3	splines	4.1.3	R Core Team. R: A language and environment for statistical computing. (2022).[36]

R 4.1.3	stargazer	5.2.3	Hlavac, M. stargazer: Well-Formatted Regression and Summary Statistics Tables. (2012).[71]
R 4.1.3	stringr	1.4.0	Wickham, H. stringr: Simple, Consistent Wrappers for Common String Operations. (2022).[72]
R 4.1.3	sys	3.4	Ooms, J. sys: Powerful and Reliable Tools for Running System Commands in R. (2017).[73]
R 4.1.3	tidyr	1.2.0	Wickham, H. et al. Welcome to the tidyverse. J. Open Source Softw. 4, 1686 (2019).[74]
R 4.1.3	tikzDevice	0.12.3.1	Stubner, R. tikzDevice: R Graphics Output in LaTeX Format. (2009).[75]
R 4.1.3	xcms	3.16.1	Smith, C. A., Want, E. J., O'Maille, G., Abagyan, R. & Siuzdak, G. XCMS: Processing mass spectrometry data for metabolite profiling using nonlinear peak alignment, matching and identification. Anal. Chem. 78, 779–787 (2006).[76]
R 4.1.3	xlsx	0.6.5	Arendt, C. xlsx: Read, Write, Format Excel 2007 and Excel 97/2000/XP/2003 Files. (2010).[77]
R 4.1.3	XML	3.99.0.9	C. R. A. N. Team, XML: Tools for Parsing and Generating XML Within R and S-Plus. (2000).[78]
R 4.1.3	xml2	1.3.3	Wickham, H. xml2: Parse XML. (2015).[79]
R 4.1.3	xtable	1.8.4	Scott, D. xtable: Export Tables to LaTeX or HTML. (2000).[80]
R 4.2.2	factoextra	1.0.7	Kassambara, A. factoextra: Extract and Visualize the Results of Multivariate Data Analyses. (2016).[81]
R 4.2.2	forcats	1.0.0	Wickham, H. forcats: Tools for Working with Categorical Variables (Factors. (2023).[82]
R 4.2.2	ggbeeswarm	0.7.1	Clarke, E. ggbeeswarm: Categorical Scatter (Violin Point. (2016).[83]
R 4.2.2	ggpmisc	0.5.2	Aphalo, P. J. ggpmisc: Miscellaneous Extensions to 'ggplot2'. (2016).[84]
R 4.2.2	ggpubr	0.6.0	Kassambara, A. ggpubr: 'ggplot2' Based Publication Ready Plots. (2016).[85]
R 4.2.2	ggsignif	0.6.4	Ahlmann-Eltze, C. ggsignif: Significance Brackets for 'ggplot2'. (2017).[86]
R 4.2.2	ggtext	0.1.2	Wiernik, B. M. ggtext: Improved Text Rendering Support for 'ggplot2'. (2020).[87]
R 4.2.2	grid	4.2.2	R Core Team. R: A language and environment for statistical computing. (2022).[36]
R 4.2.2	gridExtra	2.3	Auguie, B. gridExtra: Miscellaneous Functions for "Grid. (2010).[88]
R 4.2.2	Hmisc	4.8.0	Harrell, F. E., Jr. Hmisc: Harrell Miscellaneous. (2003).[38]
R 4.2.2	naniar	1.0.0	Tierney, N. naniar: Data Structures, Summaries, and Visualisations for Missing Data. (2017).[89]
R 4.2.2	psycho	0.6.1	Makowski, D. psycho: Efficient and Publishing-Oriented Workflow for Psychological Science. (2017).[90]
R 4.2.2	RColorBrewer	1.1.3	Neuwirth, E. RColorBrewer: ColorBrewer Palettes. R package version 1.1-3. (2002).[91]
R 4.2.2	shapes	1.2.7	Dryden, I. shapes: Statistical Shape Analysis. (2003).[92]

R 4.2.2	stats	4.2.2	R Core Team. R: A language and environment for statistical computing. (2022).[36]
R 4.2.2	svglite	2.1.1	Pedersen, T. L. svglite: An 'SVG' Graphics Device. (2015).[93]
R 4.2.2	tidyquant	1.0.6	Dancho, M. tidyquant: Tidy Quantitative Financial Analysis. (2016).[94]
R 4.2.2	tidyverse	2.0.0	Wickham, H. et al. Welcome to the tidyverse. J. Open Source Softw. 4, 1686 (2019).[74]

Supplementary Table S9: **Measurement principles for enzymatic and colorimetric assays.** All reagents were supplied by Thermo Fischer Scientific Inc.

Test	Principle	Reagents	Reference
Acetic acid	Enzymatic	Reagent 1 (buffer, D-glucose, AK, PTA, ADP-HK, G6P-DH)	984318
		Reagent 2 (NAD, ATP, CoA)	
Ammonia	Enzymatic	Reagent 1 (buffer, ADP, GLDH, clearing reagents)	984320
		Reagent 2 (NADH)	
		Reagent 3 (buffer, 2-oxoglutaramate)	
Bitterness	Colorimetry	BC system liquid	984353
		BC diluent	984354
		BC eluent	984355
Color	Colorimetry	-	-
Sugars	Enzymatic	Reagent 1 (buffer, B-Fructosidase)	984317
		Reagent 2 (buffer, ATP, NAD)	
		Reagent 3 (HK, G6P-DH)	
		Reagent 4 (Buffer, PGI)	
Glycerol	Enzymatic	Reagent 1 (buffer, D-glucose, GK, ADP-HK, G6P-DH)	984316
		Reagent 2 (buffer, NAD, ATP)	
Iron	Colorimetry	Reagent A (thiourea, guanidine buffer)	984326
		Reagent B (ascorbic acid)	
		Reagent C (ferene S, acetate buffer)	
pH	Colorimetry	Reagent 1 (pH sensitive dye)	984349
Protein	Colorimetry	Reagent total protein (NaOH, CuSO4, KI, Na2EDTA)	984328
Sulfite	Colorimetry	Reagent (buffer, DTNB)	984345
		Reagent blank (buffer)	

REFERENCES

1. American Society of Brewing Chemists. *Sensory Analysis Methods*. (American Society of Brewing Chemists, St. Paul, MN, U.S.A., 1992).
2. Koen van Gelder. Leading food retailers in Belgium by market share. https://www.statista.com/statistics/978630/leading-food-retailers-in-belgium-by-market-share/.
3. Nakatani, S. Langdetect: Language Detection Library for Java. (2010).
4. Lui, M. & Baldwin, T. langid.py: An Off-the-shelf Language Identification Tool. in *Proceedings of the ACL 2012 System Demonstrations* 25-30, (2012).
5. Bird, S., Klein, E. & Loper, E. *Natural Language Processing with Python: Analyzing Text with the Natural Language Toolkit*. (2009).
6. Kouzis-Loukas, D. *Learning Scrapy*. (2016).
7. Anaconda Software Distribution. Anaconda Documentation. (2023).
8. Joblib Development Team. Joblib: running Python functions as pipeline jobs. (2020).
9. Harris, C. R. *et al.* Array programming with NumPy. *Nature* **585**, 357–362 (2020).
10. McKinney, W. & al. Data structures for statistical computing in python. in *Proceedings of the 9th Python in Science Conference* vol. 445 51–56 (2010).
11. Pedregosa, F. *et al.* Scikit-learn: Machine learning in Python. *J. Mach. Learn. Res.* **12**, 2825–2830 (2011).
12. Waskom, M. *et al.* Seaborn. (2017).
13. Lundberg, S. M., Erion, G. & Chen, H. From local explanations to global understanding with explainable AI for trees. *Nat. Mach. Intell.* **2**, 56–67 (2020).
14. Chen, T. & Guestrin, C. XGBoost: A Scalable Tree Boosting System. in *Proceedings of the 22nd ACM SIGKDD International Conference on Knowledge Discovery and Data Mining* 785–794 (New York, NY, USA, 2016). doi:10.1145/2939672.2939785.
15. Plate, T. abind: Combine Multidimensional Arrays. (2003).
16. Liland, K. H. baseline: Baseline Correction of Spectra. (2011).
17. Couch, S. broom: Convert Statistical Objects into Tidy Tibbles. (2014).
18. Urbanek, S. Cairo: R Graphics Device using Cairo Graphics Library for Creating High-Quality. (2007).
19. Fox, J. car: Companion to Applied Regression. (2001).
20. Dietze, M. caTools: Tools: Moving Window Statistics, GIF, Base64, ROC AUC, etc. (2005).
21. Brock, G., Pihur, V., Datta, S. & Datta, S. clValid: An R Package for Cluster Validation. *J. Stat. Softw.* **25**, 1–22 (2008).
22. Ng, P. & Maechler, M. A Fast and Efficient Implementation of Qualitatively Constrained Quantile Smoothing Splines. *Stat. Model.* **7**, 315–328 (2007).
23. Keitt, T. colorRamps: Builds Color Tables. R package version 2.3. (2007).
24. Dowle, M. data.table: Extension of `data.frame`. R package version 1.14.2. (2006).
25. Wickham, H., Hester, J., Chang, W. & Bryan, J. devtools: Tools to Make Developing R Packages Easier. (2022).
26. Daniel, F. doParallel: Foreach Parallel Adaptor for the ‘parallel’ Package. (2011).
27. Daniel, F. doSNOW: Foreach Parellel Adaptor for the ‘snow’ Package. (2022).
28. Wickham, H., François, R., Henry, L., Müller, K. & Vaughan, D. dplyr: A Grammar of Data Manipulation. (2023).
29. Wenseleers, T. export: Streamlined Export of Graphs and Data Tables. (2018).
30. Guha, R. fingerprint: Functions to Operate on Binary Fingerprint Data. (2006).
31. Gruen, B. flexmix: Flexible Mixture Modeling. (2003).
32. Gohel, D. flextable: Functions for Tabular Reporting. (2017).
33. Hennig, C. fpc: Flexible Procedures for Clustering. (2003).
34. Wickham, H. *ggplot2: Elegant Graphics for Data Analysis*. (2016).
35. Friedman, J., Hastie, T. & Tibshirani, R. Regularization Paths for Generalized Linear Models via Coordinate Descent. *J. Stat. Softw.* **33**, 1–22 (2010).
36. R Core Team. R: A language and environment for statistical computing. R Foundation for Statistical Computing (2022).
37. Verzani, J. gWidgets: gWidgets API for building toolkit-independent, interactive GUIs. (2014).
38. Harrell, F. E., Jr. Hmisc: Harrell Miscellaneous. (2003).

39. Wickham, H. httr: Tools for Working with URLs and HTTP. (2023).
40. Venables, W. N. & Ripley, B. D. *Modern Applied Statistics with S*. (New York, 2002).
41. Maechler, M. Matrix: Sparse and Dense Matrix Classes and Methods. (2000).
42. Ekstrm, C. T. MESS: Miscellaneous Esoteric Statistical Scripts. (2012).
43. Wehrens, R., Weingart, G. & Mattivi, F. metaMS: An open-source pipeline for GC-MS-based untargeted metabolomics. *J. Chromatogr. B* **966**, 109–116 (2014).
44. Henningsen, A. miscTools: Miscellaneous Tools and Utilities. (2009).
45. Filzmoser, P. mvoutlier: Multivariate Outlier Detection Based on Robust Methods. (2004).
46. Chambers, C. M. *et al.* A cross-platform toolkit for mass spectrometry and proteomics. *Nat Biotech* **30**, 918–920 (2012).
47. Pierce, D. ncdf4: Interface to Unidata netCDF (Version 4 or Earlier. (2010).
48. Ripley, B. nnet: Feed-Forward Neural Networks and Multinomial Log-Linear Models. (2009).
49. Mullen, K. nnls: The Lawson-Hanson algorithm for non-negative least squares(NNLS. (2007).
50. Gohel, D. officer: Manipulation of Microsoft Word and PowerPoint Documents. (2023).
51. Schauberger, P. & Walker, A. openxlsx: Read, Write and Edit xlsx Files. (2022).
52. Dodder, N. OrgMassSpecR: Organic Mass Spectrometry. (2010).
53. Solymos, P. pbapply: Adding Progress Bar to '*apply' Functions. (2010).
54. Kolde, R. pheatmap: Pretty Heatmaps. (2010).
55. Wickham, H. The Split-Apply-Combine Strategy for Data Analysis. *J. Stat. Softw.* **40**, 1–29 (2011).
56. Meyer, D. proxy: Distance and Similarity Measures. (2007).
57. Wehrens, R. ptw: Parametric Time Warping. (2009).
58. Koenker, R. quantreg: Quantile Regression. (2009).
59. Bengtsson, H. R.utils: Various Programming Utilities. (2005).
60. Eddelbuettel, D. Rcpp: Seamless R and C++ Integration. R package version 1.0.8.3. (2008).
61. C.R.A.N. Team. RCurl: General Network (HTTP/FTP/...) Client Interface for R. (2004).
62. Wickham, H. reshape: Flexibly Reshape Data. (2005).
63. Murdoch, D. rgl: 3D Visualization Using OpenGL. R package version 0.108.3. (2004).
64. Lawrence, M. RGtk2: R Bindings for Gtk 2.8.0 and Above. (2018).
65. Urbanek, S. rJava: Low-Level R to Java Interface. (2005).
66. Couture-Beil, A. rjson: JSON for R. (2007).
67. Stravs, M. A., Schymanski, E. L., Singer, H. & Hollender, J. Automatic Recalibration and Processing of Tandem Mass Spectra using Formula Annotation. *J. Mass Spectrom.* **48**, 188 (2013).
68. Gohel, D. rvg: R Graphics Devices for Vector Graphics Output. (2016).
69. Ligges, U. signal: Signal Processing. (2006).
70. Tierney, L. snow: Simple Network of Workstations. (2003).
71. Hlavac, M. stargazer: Well-Formatted Regression and Summary Statistics Tables. (2012).
72. Wickham, H. stringr: Simple, Consistent Wrappers for Common String Operations. (2022).
73. Ooms, J. sys: Powerful and Reliable Tools for Running System Commands in R. (2017).
74. Wickham, H. *et al.* Welcome to the tidyverse. *J. Open Source Softw.* **4**, 1686 (2019).
75. Stubner, R. tikzDevice: R Graphics Output in LaTeX Format. (2009).
76. Smith, C. A., Want, E. J., O'Maille, G., Abagyan, R. & Siuzdak, G. XCMS: Processing mass spectrometry data for metabolite profiling using nonlinear peak alignment, matching and identification. *Anal. Chem.* **78**, 779–787 (2006).
77. Arendt, C. xlsx: Read, Write, Format Excel 2007 and Excel 97/2000/XP/2003 Files. (2010).
78. C.R.A.N. Team. XML: Tools for Parsing and Generating XML Within R and S-Plus. (2000).
79. Wickham, H. xml2: Parse XML. (2015).
80. Scott, D. xtable: Export Tables to LaTeX or HTML. (2000).
81. Kassambara, A. factoextra: Extract and Visualize the Results of Multivariate Data Analyses. (2016).
82. Wickham, H. forcats: Tools for Working with Categorical Variables (Factors). (2023).
83. Clarke, E. ggbeeswarm: Categorical Scatter (Violin Point. (2016).

84. Aphalo, P. J. ggpmisc: Miscellaneous Extensions to ‘ggplot2’. (2016).
85. Kassambara, A. ggpubr: ‘ggplot2’ Based Publication Ready Plots. (2016).
86. Ahlmann-Eltze, C. ggsignif: Significance Brackets for ‘ggplot2’. (2017).
87. Wiernik, B. M. ggtext: Improved Text Rendering Support for ‘ggplot2’. (2020).
88. Auguie, B. gridExtra: Miscellaneous Functions for "Grid. Graphics (2010).
89. Tierney, N. naniar: Data Structures, Summaries, and Visualisations for Missing Data. (2017).
90. Makowski, D. psycho: Efficient and Publishing-Oriented Workflow for Psychological Science. (2017).
91. Neuwirth, E. RColorBrewer: ColorBrewer Palettes. R package version 1.1-3. (2002).
92. Dryden, I. shapes: Statistical Shape Analysis. (2003).
93. Pedersen, T. L. svglite: An ‘SVG’ Graphics Device. (2015).
94. Dancho, M. tidyquant: Tidy Quantitative Financial Analysis. (2016).
