# Supplementary material for: Predicting and improving complex beer flavor through machine learning
Source: Nat Commun. 2024 Mar 26;15:2368. doi: 10.1038/s41467-024-46346-0 (PMC10966102; doi:10.1038/s41467-024-46346-0)
Supplement: Supplementary file 3 — Description of Additional Supplementary Files [file 41467_2024_46346_MOESM3_ESM.pdf]

Description of Additional Supplementary Files

File Name: Supplementary Data 1
Description: Chemical measurements for all 250 beer samples.

File Name: Supplementary Data 2
Description: Spearman correlation scores between all the chemical parameters.

File Name: Supplementary Data 3
Description: Tasting sheet used by the trained tasting panel during sensory analysis.

File Name: Supplementary Data 4
Description: Sensory analysis results for all 250 beer samples.

File Name: Supplementary Data 5
Description: Spearman correlation scores between all the sensory parameters.

File Name: Supplementary Data 6
Description: Spearman correlations between the chemical and sensory parameters.

File Name: Supplementary Data 7
Description: Results from RateBeer review text mining for sensory descriptors for all 250 beer samples.
