# Supplementary material for: Predicting and improving complex beer flavor through machine learning
Source: Nat Commun. 2024 Mar 26;15:2368. doi: 10.1038/s41467-024-46346-0 (PMC10966102; doi:10.1038/s41467-024-46346-0)
Supplement: Supplementary file 11 — Reporting Summary [file 41467_2024_46346_MOESM11_ESM.pdf]

nature portfolio

Corresponding author(s): Kevin J. Verstrepen

Last updated by author(s): Feb 13, 2024

Reporting Summary

Nature Portfolio wishes to improve the reproducibility of the work that we publish. This form provides structure for consistency and transparency in reporting. For further information on Nature Portfolio policies, see our Editorial Policies and the Editorial Policy Checklist.

Statistics

For all statistical analyses, confirm that the following items are present in the figure legend, table legend, main text, or Methods section.

n/a	Confirmed	
☐	☒	The exact sample size (*n*) for each experimental group/condition, given as a discrete number and unit of measurement
☐	☒	A statement on whether measurements were taken from distinct samples or whether the same sample was measured repeatedly
☐	☒	The statistical test(s) used AND whether they are one- or two-sided<br>*Only common tests should be described solely by name; describe more complex techniques in the Methods section.*
☒	☐	A description of all covariates tested
☐	☒	A description of any assumptions or corrections, such as tests of normality and adjustment for multiple comparisons
☐	☒	A full description of the statistical parameters including central tendency (e.g. means) or other basic estimates (e.g. regression coefficient) AND variation (e.g. standard deviation) or associated estimates of uncertainty (e.g. confidence intervals)
☒	☐	For null hypothesis testing, the test statistic (e.g. *F*, *t*, *r*) with confidence intervals, effect sizes, degrees of freedom and *P* value noted<br>*Give P values as exact values whenever suitable.*
☒	☐	For Bayesian analysis, information on the choice of priors and Markov chain Monte Carlo settings
☒	☐	For hierarchical and complex designs, identification of the appropriate level for tests and full reporting of outcomes
☐	☒	Estimates of effect sizes (e.g. Cohen's *d*, Pearson's *r*), indicating how they were calculated

Our web collection on statistics for biologists contains articles on many of the points above.

Software and code

Policy information about availability of computer code

Data collection	Python (version 3.6) was used for gathering RateBeer review data<br>R (version 4.1.3) was used for processing of GCMS spectra
Data analysis	Python (version 3.9.16) was used for machine learning modeling<br>R (version 4.2.2) was used for figure generation and data analysis

For manuscripts utilizing custom algorithms or software that are central to the research but not yet described in published literature, software must be made available to editors and reviewers. We strongly encourage code deposition in a community repository (e.g. GitHub). See the Nature Portfolio guidelines for submitting code & software for further information.

Data

Policy information about availability of data

All manuscripts must include a data availability statement. This statement should provide the following information, where applicable:

- Accession codes, unique identifiers, or web links for publicly available datasets
- A description of any restrictions on data availability
- For clinical datasets or third party data, please ensure that the statement adheres to our policy

The data that support the findings of this work are available in the Supplementary files and have been deposited to Zenodo under accession code 1065370495. The

RateBeer scores data are under restricted access, they are not publicly available as they are property of RateBeer (ZX Ventures, USA). Access can be obtained from the authors upon reasonable request and with permission of RateBeer (ZX Ventures, USA).

Research involving human participants, their data, or biological material

Policy information about studies with human participants or human data. See also policy information about sex, gender (identity/presentation), and sexual orientation and race, ethnicity and racism.

Reporting on sex and gender	Previous work established differences between the sexes in terms of flavor sensitivity and appreciation. In this work, the influence of sex was not considered. Instead, results from different panelists were averaged, both for our trained panel (56% male, 44% female) and the RateBeer reviews (70% male and 30% female for the entire RateBeer dataset), for which no sex information was available for individual reviews. Grouping tasters based on sex and incorporating a sex identifier might therefore lead to improved models, although we do not expect that this would affect our general conclusions.
Reporting on race, ethnicity, or other socially relevant groupings	No groupings were made based on race, ethnicity or any social groups.
Population characteristics	age: 22-42, mean: 29<br>sex: 56% male, 44% female<br>nationality: 7 different countries
Recruitment	Participants were recruited from our own research group. All participants volunteered, were informed and underwent a screening to evaluate their sensory performance (triangle tests).
Ethics oversight	Social and Societal Ethics Committee of the KU Leuven

Note that full information on the approval of the study protocol must also be provided in the manuscript.

Field-specific reporting

Please select the one below that is the best fit for your research. If you are not sure, read the appropriate sections before making your selection.

☒ Life sciences ☐ Behavioural & social sciences ☐ Ecological, evolutionary & environmental sciences

For a reference copy of the document with all sections, see nature.com/documents/nr-reporting-summary-flat.pdf

Life sciences study design

All studies must disclose on these points even when the disclosure is negative.

Sample size	The beer dataset comprised 250 beer samples.<br>The trained tasting panel consisted of 16 people.<br>The number of RateBeer reviewers varied per sample.
Data exclusions	RateBeer reviewers who had rated less than 100 different beers (in total, not just our set of 250) were excluded.<br>No other data were excluded from the analyses.
Replication	All chemical analyses were repeated in biological duplicate.
Randomization	Randomization is not relevant for most of our study.<br>In the validation tastings, the order in which samples were tasted, was randomized.
Blinding	Participants were blinded during the tasting sessions.

Reporting for specific materials, systems and methods

We require information from authors about some types of materials, experimental systems and methods used in many studies. Here, indicate whether each material, system or method listed is relevant to your study. If you are not sure if a list item applies to your research, read the appropriate section before selecting a response.

Materials & experimental systems

n/a	Involved in the study	
☒	☐	Antibodies
☒	☐	Eukaryotic cell lines
☒	☐	Palaeontology and archaeology
☒	☐	Animals and other organisms
☒	☐	Clinical data
☐	☒	Dual use research of concern
☒	☐	Plants

Methods

n/a	Involved in the study	
☒	☐	ChIP-seq
☒	☐	Flow cytometry
☒	☐	MRI-based neuroimaging

Dual use research of concern

Policy information about dual use research of concern

Hazards

Could the accidental, deliberate or reckless misuse of agents or technologies generated in the work, or the application of information presented in the manuscript, pose a threat to:

No	Yes	
☐	☒	Public health
☒	☐	National security
☒	☐	Crops and/or livestock
☒	☐	Ecosystems
☒	☐	Any other significant area

Hazards: Deliberate misuse of the technologies described in this work could lead to the design and production of more addictive, harmful substances.

For examples of agents subject to oversight, see the United States Government Policy for Institutional Oversight of Life Sciences Dual Use Research of Concern.

Experiments of concern

Does the work involve any of these experiments of concern:

No	Yes	
☒	☐	Demonstrate how to render a vaccine ineffective
☒	☐	Confer resistance to therapeutically useful antibiotics or antiviral agents
☒	☐	Enhance the virulence of a pathogen or render a nonpathogen virulent
☒	☐	Increase transmissibility of a pathogen
☒	☐	Alter the host range of a pathogen
☒	☐	Enable evasion of diagnostic/detection modalities
☒	☐	Enable the weaponization of a biological agent or toxin
☒	☐	Any other potentially harmful combination of experiments and agents

Precautions and benefits

Biosecurity precautions: No biosecurity precautions are required.

Biosecurity oversight: No biosecurity oversight was involved in this work.

Benefits: The technology in this work can be employed to improve flavor, and possibly other sensory responses. Its ability to enhance the flavor, and thus appreciation and consumption, of healthy or ecological foods could help with numerous food and nutrition related issues that our world is facing, such as:
- Obesity and the myriad of related health concerns, through improving the flavor of sugar-free or low calorie foods.
- Alcohol consumption and the related health concerns, by improving the flavor and experience of non-alcoholic alternatives.
- Meat consumption and its ecological impact, by improving the flavor of plant-based meat alternatives.

Communication benefits: We believe the benefits of this technology, when contributing to better appreciated, healthier and ecological foods, outweighs the risk of it being used to produce more addictive or harmful products.
The growing consumer awareness of consumption is already pushing manufacturers to produce healthier and ecological products.
We hope that this ensures our technology is used predominantly for the benefit of humanity, and not for harmful intent.

Plants

Seed stocks	*Report on the source of all seed stocks or other plant material used. If applicable, state the seed stock centre and catalogue number. If plant specimens were collected from the field, describe the collection location, date and sampling procedures.*
Novel plant genotypes	*Describe the methods by which all novel plant genotypes were produced. This includes those generated by transgenic approaches, gene editing, chemical/radiation-based mutagenesis and hybridization. For transgenic lines, describe the transformation method, the number of independent lines analyzed and the generation upon which experiments were performed. For gene-edited lines, describe the editor used, the endogenous sequence targeted for editing, the targeting guide RNA sequence (if applicable) and how the editor was applied.*
Authentication	*Describe any authentication procedures for each seed stock used or novel genotype generated. Describe any experiments used to assess the effect of a mutation and, where applicable, how potential secondary effects (e.g. second site T-DNA insertions, mosiacism, off-target gene editing) were examined.*
